# Research with radiation and radioisotopes to better understand plant physiology and agricultural consequences of radioactive contamination from the Fukushima Daiichi nuclear accident

**DOI:** 10.1007/s10967-016-5148-z

**Published:** 2017-01-04

**Authors:** Tomoko M. Nakanishi

**Affiliations:** Graduate School of Agricultural and Life Sciences, The University of Tokyo, 1-1-1, Yayoi, Bunkyo-Ku, Tokyo 113-8657 Japan

**Keywords:** Neutron imaging, Water imaging and measurement, Radioisotope, Real-time radioisotope imaging system, Plant physiology, Fukushima nuclear accident, Agricultural impact of contamination

## Abstract

Research carried out by me and my group over the last almost four decades are summarized here. The main emphasis of my work was and continues to be on plant physiology using radiation and radioisotopes. Plants live on water and inorganic elements. In the case of water, we developed neutron imaging methods and produced ^15^O-labeled water (half-life 2 min) and applied them to understand water circulation pattern in the plant. In the case of elements, we developed neutron activation analysis methods to analyze a large number of plant tissues to follow element specific distribution. Then, we developed real-time imaging system using conventional radioisotopes for the macroscopic and microscopic observation of element movement. After the accident in Fukushima Daiichi nuclear power plant, we, the academic staff of Graduate School, have been studying agricultural effects of radioactive fallout; the main results are summarized in two books published by Springer.

## Introduction

First of all, I would like to express my sincere thanks to the members of Hevesy Medal Award Selection Panel 2016 as well as to all the people who supported me for the Hevesy Medal Award. I would like to summarize in this paper the kind of research I have been doing in my life.

After determining the half-lives of long-lived nuclides, namely ^91^Nb and ^92^Nb for my PhD thesis, I have been targeting plant physiology for many years and applying radiochemical approaches. Though plants live on only inorganic elements and water, little is known about the distribution or movement of these and water in a living plant. For example, photosynthesis is known to produce sucrose out of CO_2_ and water but water itself has not been gathering the attention. Water was simply granted to exist there but is playing an important role for the chemical process of the energy conversion, form light to chemical energy. However, we do not know how water is absorbed and transferred in the plant.

Therefore, my first interest was water, in particular how water is distributed and move within a living plant as well as its absorption from roots. To study water distribution in plants, neutron beam was applied which produced water-specific image of the plant. The neutron beam allowed imaging not only water itself but also the morphological development of the plant tissue which was not visualized earlier, such as seed formation in pods or roots imbedded in soil. Then, ^15^O labeled water was used to trace the water movement in detail and we found that tremendous amount of water was always flowing out from xylem and re-entered the xylem again, indicating that there is a circulation of water flow in the stem of a soybean plant.

Element was another target of my research. There are 17 essential elements for plant growth, but little is known about the overall accumulation or movement manner of the elements. Activation analysis was performed for a large number of plant tissues and the element-specific accumulation pattern in the plant was found; and this pattern was maintained in the same way throughout the developmental stage. When flowering was induced, Mg-specific distribution pattern disappeared which led later to develop the production of the radioactive magnesium tracer, ^28^Mg (half-life: 21 h).

Since each element showed its specific distribution pattern in a plant, next step was the development of real-time imaging of the elements. Though imaging using positron emitters has been developed especially in medical field known as PET (Positron Emission Tomography), its resolution cannot be less than mm because of the relatively high positron energy. In the case of fluorescence imaging, imaging under light condition is not possible and the amount of the element in the image could not be measured. Therefore, the real-time RI imaging system was developed by us using not only gamma-ray but also beta emitters which are commercially available so that other people can also use them. We have been successful in developing the systems both for macroscopic and microscopic imaging. The real-time movement of the elements can now be visualized and analyzed using ^14^C, ^22^Na, ^28^Mg, ^32^P, ^33^P, ^35^S, ^42^K, ^45^Ca, ^54^Mn, ^55^Fe, ^59^Fe,^65^Zn, ^86^Rb, ^109^Cd, ^137^Cs, etc. The image of ^14^CO_2_ gas fixation provided that the photosynthate was moved quickly to produce the meristem of the root tip.

After Fukushima nuclear accident, our group studied the agricultural consequences of radioactive contamination from the Fukushima Daiichi reactors. I was able to edit two English books summarizing our data, published by Springer. The on-line version of the first book was accessed more than 50,000 times a year and the third book is now going to be published next year.

A brief survey of many research projects carried out over the years by me and my group is given below.

## Research topics: methodology, results and discussion

**Fig. 1:**
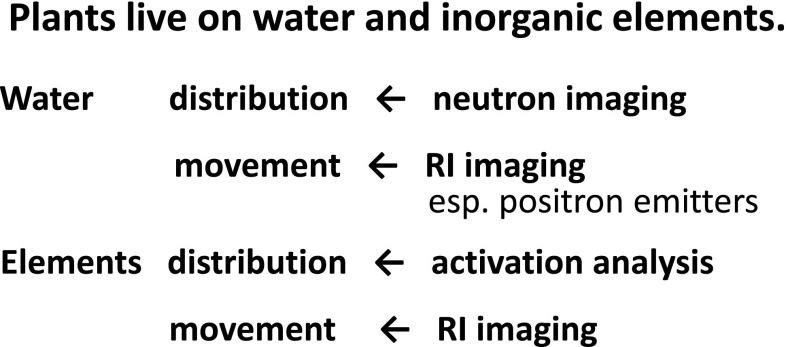
Application of radiation or radioisotopes to plant study

Since plants live on water and inorganic elements, the applied radiation or radioisotope method used in presented in Fig. 1.

### Neutron beam imaging: water distribution [1–27]

#### Flower, wood disk and seeds

Neutron beam was applied to observe water-specific image in a living plant, since neutron imaging provided the highest resolution yet attainable for water in tissue. With high specificity for water, neutron beam could image water movement in seeds or in roots imbedded in soil as well as in wood disks and meristems during the development. Through neutron image analysis, we were able to analyze the activity of intact cells or tissue.

**Fig. 2:**
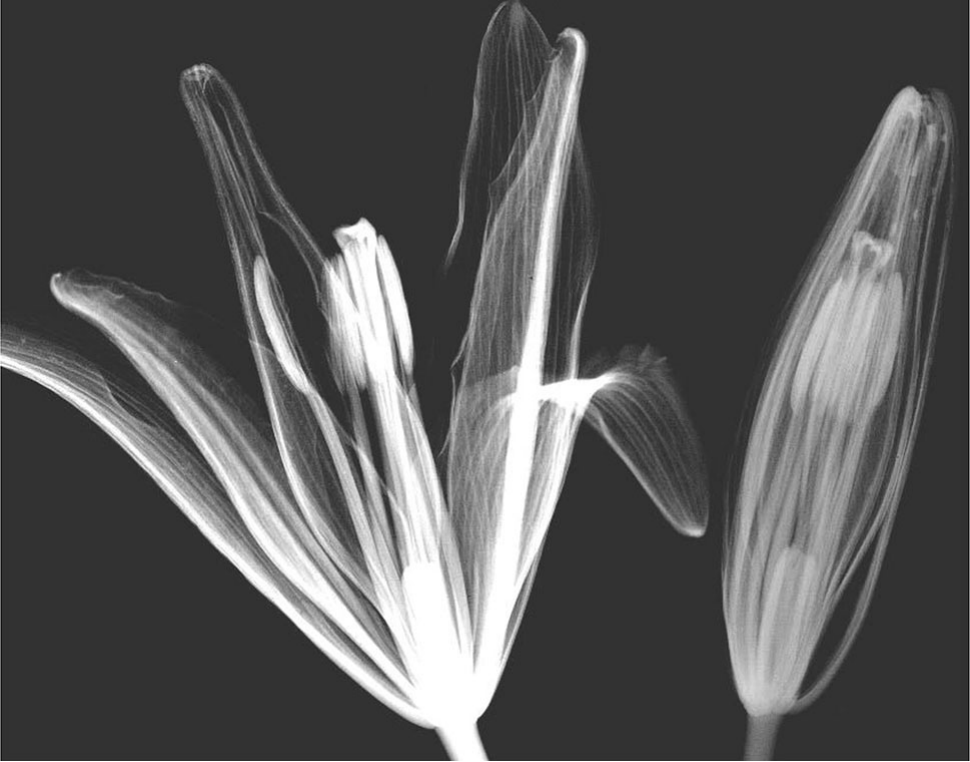
Water image of lily flower and bud by neutron beam. *Note* The degree of penetration of the neutron beam is highly dependent on the amount of water in the sample. The *whiter* part is the place where water content is higher; so the neutron beam could not penetrate the sample well; therefore, the exposure of the X-ray film behind the plants was low, which resulted in whiter image. Through calculation, the amount of water in tissue can be obtained

Since more than 80% of the living plant is consisted of water, water image indicates the tissue image itself. Figure 2 is one of the examples of the water-specific image of lily and from this image we can estimate how the pistil and stamen inside the bud are developing. Similar to the flower bud, the seed formation inside the pod became visible. In the case of agricultural industry, to create the sterile plant which does not develop the seed, is one of the important feature to be able to provide the seeds every year. (not clear what the author wants to say in the last sentence).

**Fig. 3:**
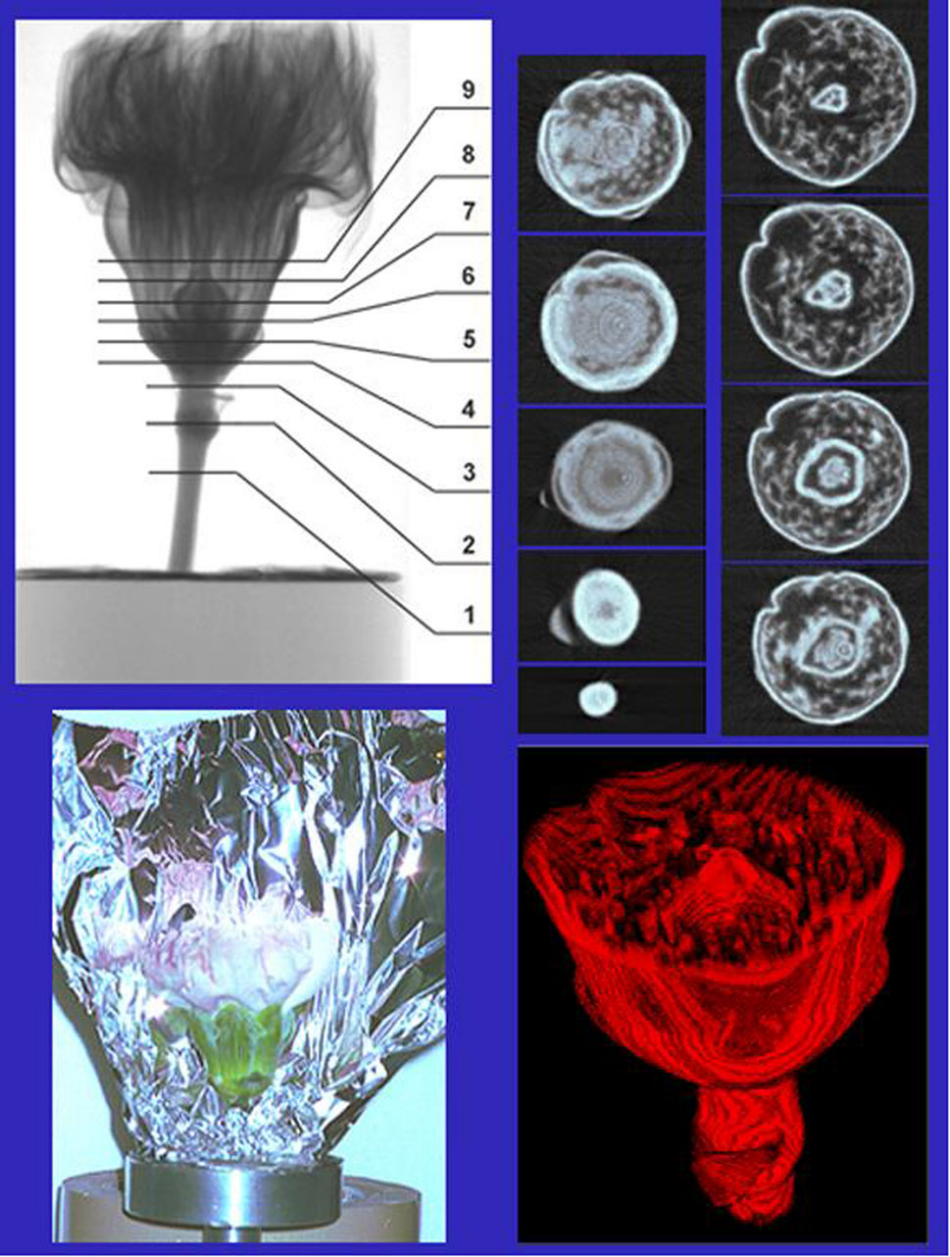
Three dimensional water image of carnation flower. *Note* Carnation flower was wrapped with an aluminum foil and was rotated during the neutron beam irradiation. One degree by one degree the sample was rotated and at each angle the neutron projection image was taken. In this case, 180 projection images were taken while rotating the sample till 180°. Out of 180 projection images, one line at the same height of the images were taken out and the transverse section image was constructed by computer. When all the cross section images constructed every 50 μm interval at height were obtained, they were piled up to construct the three dimensional water image. An example of the spatial image of the lower part of the flower (2 cm) is shown. Lower part was selected so that the inner part could be clearly distinguished

Another requirement from flower industry is to keep the flowering time of the cult flowers longer. One of the solutions is to change the viscosity of water supplied to the cut flowers. When the xenon gas was dissolved in water under pressure and supplied to the cut flowers, it was found to slower the senescence, which was studied using a carnation flower. To analyze the effect of the water prepared, spatial distribution of the water within the flower was constructed (Fig. 3). When the water decreasing part in flower was visualized, keeping water around the ovule was crucial and applying water with high viscosity, such as Xe gas dissolved water, was found to be effective. In the case of roses, there was a specific problem called “bent neck” phenomenon. When the flower is bent, it never comes back again to the straight position even with fresh supply of plenty of water. The neutron beam imaging suggested that there are two types of tissues in the stem below the flower, which allows water absorption after draught condition.

**Fig. 4:**
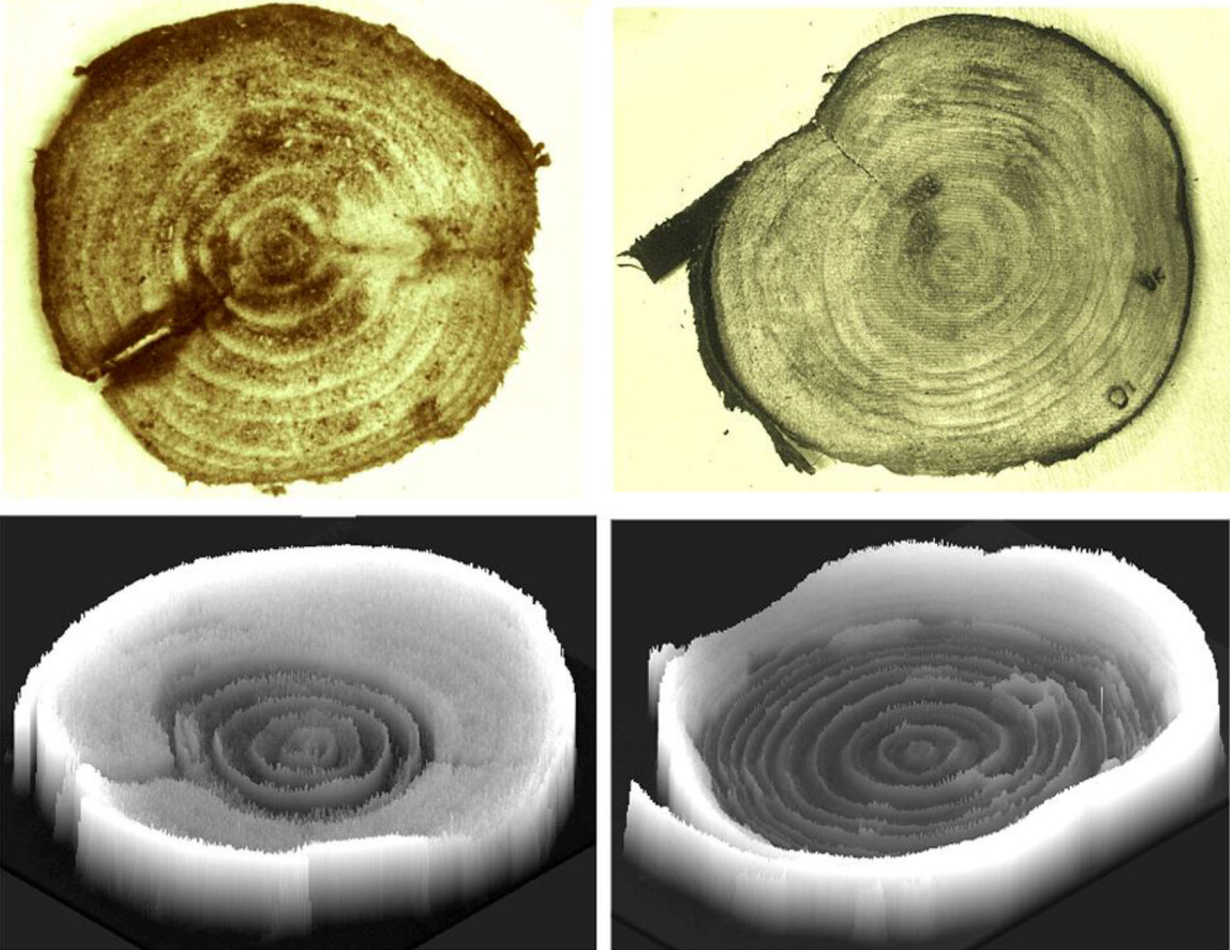
Water distribution of acacia and Japanese cypress. *Note* The upper two images are pictures and the lower two images are neutron beam images. *Left* acacia, *right* Japanese cypress. Wood discs, 1 cm in width, were prepared just before neutron beam irradiation. The whiteness of the image obtained in an X-ray film after irradiation corresponded to the water image; therefore, the 2-dimensional image was converted to a spatial one where the height was the degree of the whiteness representing the water content. In the case of acacia, heartwood formation could not be confirmed form the picture because it did not change any color. However, the lower picture showed clear difference of the water distribution between heartwood and sapwood parts. Water image of the Japanese cypress showed high amount of water in only outer part of the wood and small amount of water was distributed along the annual ring in inner part

**Fig. 5:**
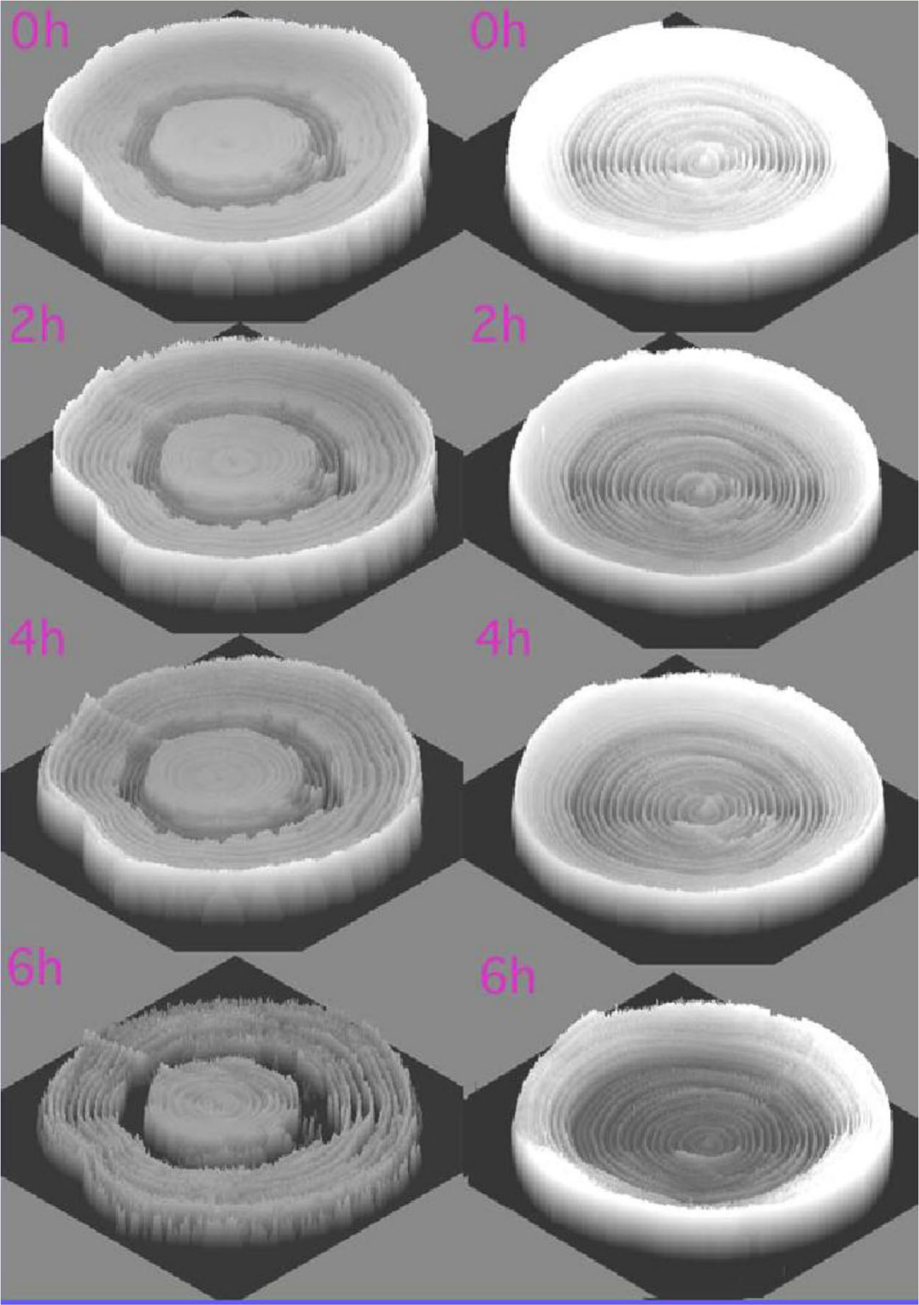
Water image of cedar tree during drying process. *Note* Wood disks of cedar tree, 1 cm in thickness, were taken out every 2 h during the drying process and neutron image was obtained. *Left* high water content in heartwood; *right* low water content in heartwood. When the initial water content at heartwood is high, it takes longer time to reduce the amount of water compared that with lower water content

Several kinds of tree were downed and water distribution within the bark disk was investigated. The water in the bark is liable to evaporate fast from the cut surface, therefore, the trees were cut down as a log on the day of neutron irradiation and was further cut to the wood disk just before the imaging. In the case of acacia, although the heartwood formation was not observed from outside, water distribution showed as if heartwood was developed where water amount was very low. The color of the Japanese cypress did not show any heartwood formation like acacia, from the water distribution image of the disk (Fig. 4). Lumber of cedar trees are very popular materials in Japan for constructing houses or making furniture but water amount at heartwood cannot be known until downed. Even the same kind of the trees growing in the neighbor, the amount of the water at heartwood is different. When the water amount in the heartwood is high, it is hardly possible to remove all of the water from the log by drying process and remaining water causes distortion or cracks after construction. When neutron beam was applied, water was found to distribute according to the annual ring inside the cedar disk. Neutron images were taken periodically during the drying process and water amount and distribution of the disk were analyzed (Fig. 5).

**Fig. 6:**
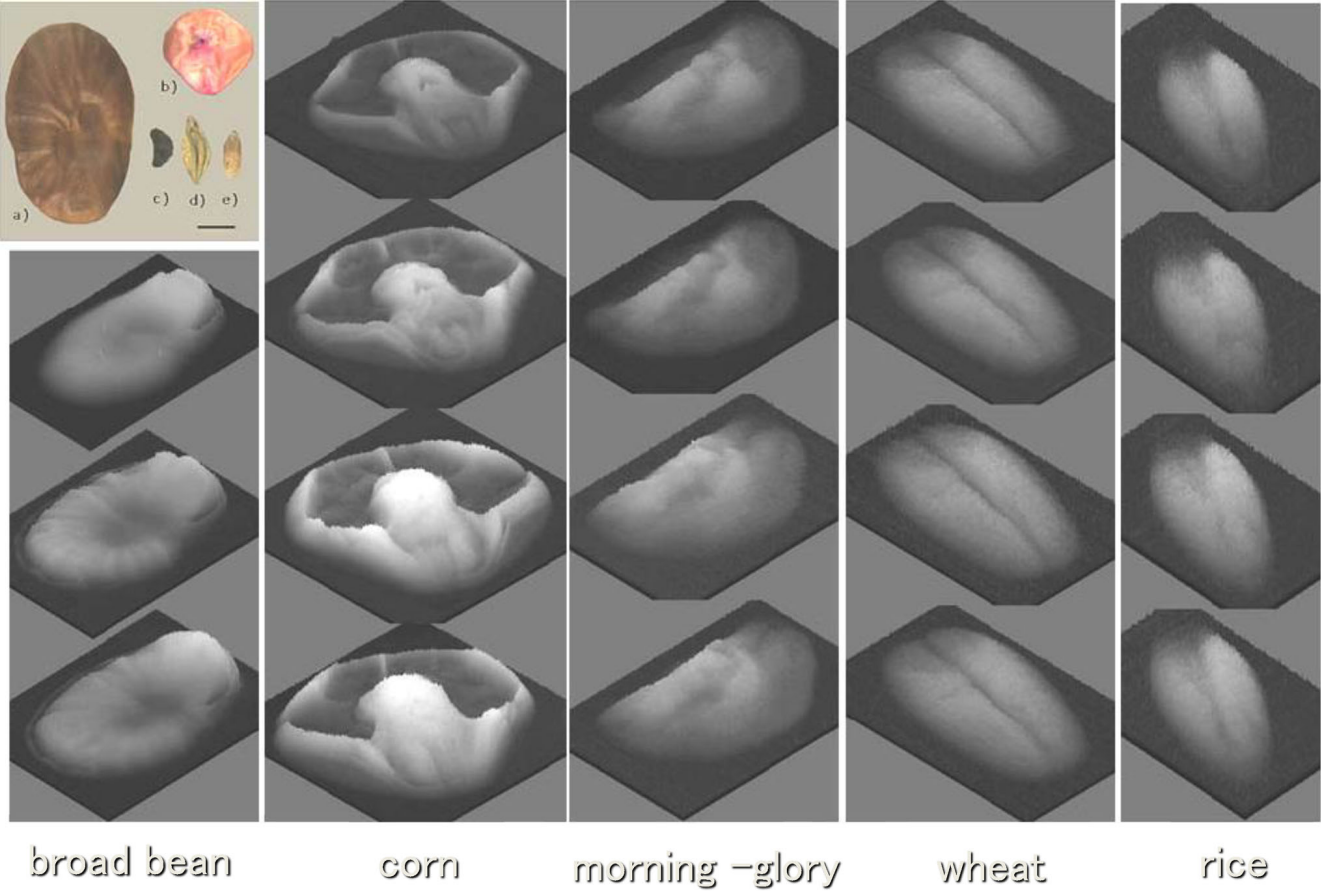
Water absorption process of seeds. Five kinds of seeds, broad bean, corn, morning-glory, wheat and rice were placed in water and every 2 h they were taken out from the water and neutron imaging was performed. *Upper left* picture of five seeds. *Bar* 5 mm. The distribution of water after absorption was not uniform in all the seeds. Water was preferentially accumulated at embryo site in seeds

The water absorption in seeds is still not known well, though it was estimated that the seeds do not absorb and accumulate water homogeneously. Neutron beam imaging provided some clue to this question. Figure 6 shows the water image in 5 different seeds, broad bean, corn, morning-glory, wheat and rice. They were put in water container and they were taken out every 2 h to take water images. It was shown that water was accumulated to the embryo, meristem part, after absorption. The specific accumulation of water in the seed was well visualized for the corn seed, where water was hardly accumulated to the other parts but to embryo (Fig. 6).

#### Roots imbedded in soil

**Fig. 7:**
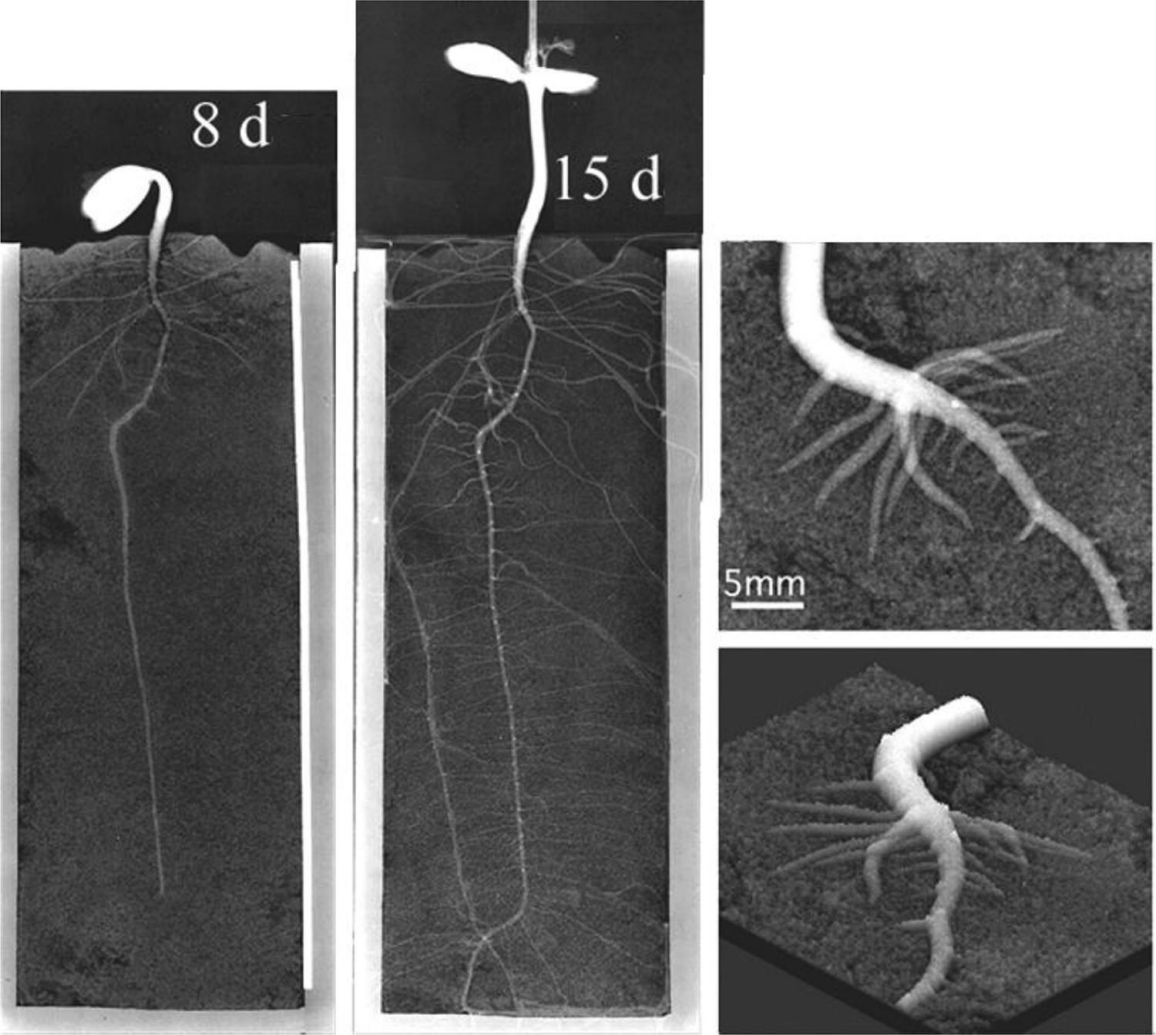
Neutron image of soybean root imbedded in soil. *Note* A soybean plant was grown in a thin (3 mm) aluminum box packed with soil containing 15% water. After 8 and 15 days, neutron images were taken. Since the water concentration in root was higher than that in the soil, morphological development of the root as well as water movement was analyzed from the image. *Upper right* magnification of the root when side root emerged to develop; *lower right* conversion of the upper image to 3 dimensional one, where the water amount was employed as height

**Fig. 8:**
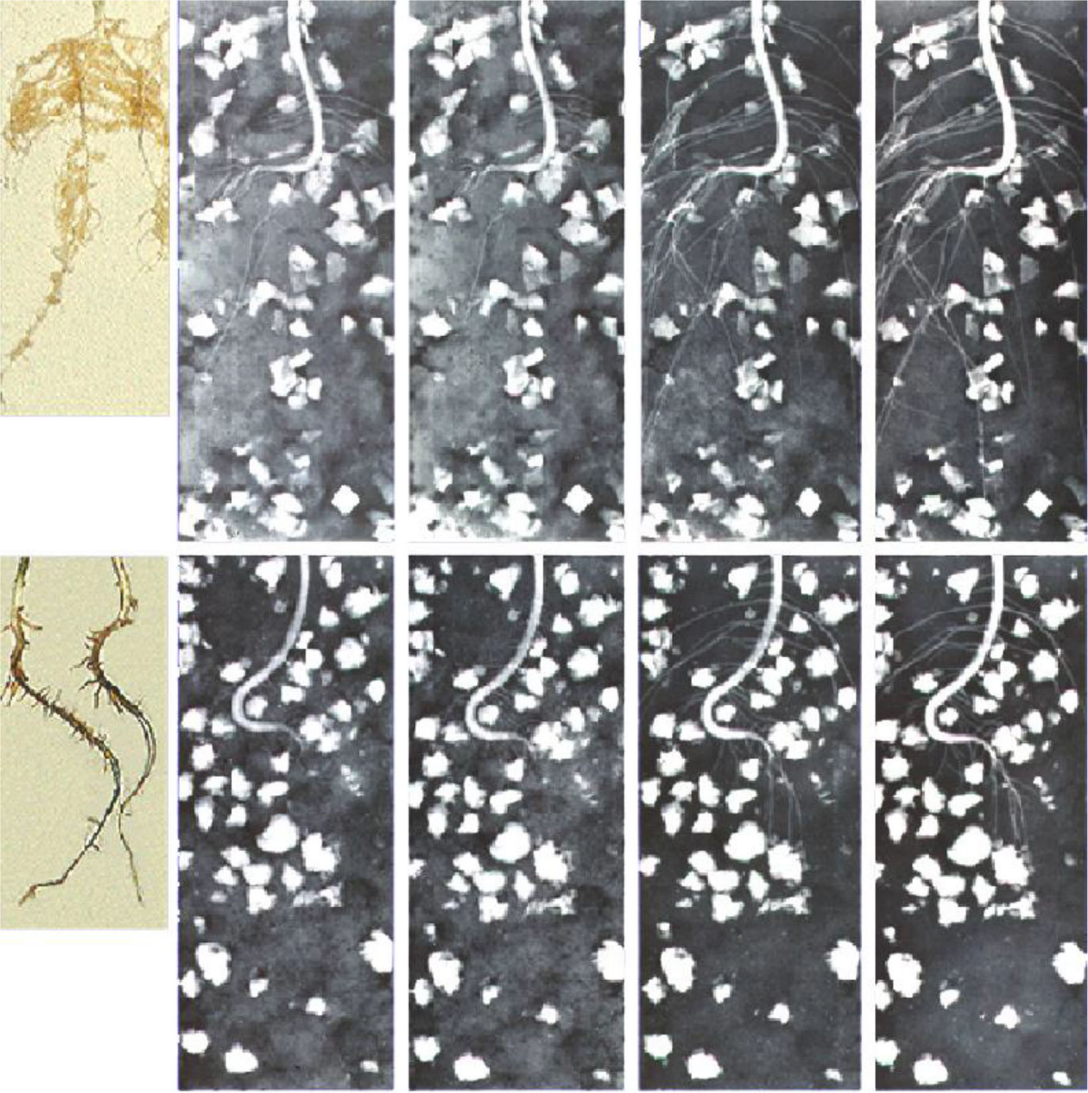
Soybean root development with water absorbing polymer in soil. Two kinds of polymers, polyvinyl-alcohol polymer (*top*) and poly-acrylic polymer (*bottom*) were swelled in water solution and mixed to the soil where soybean plants were grown. Samples were periodically taken out from phytotron and neutron image was taken. In the case of poly-acrylic polymer, the water absorbed by the polymer was not supplied to the plant and the color of the soil darkened with time indicating that the plant was absorbing the water only from the soil. While the polyvinyl-alcohol polymer supplied water to the plants and the image was gradually decreasing. *Left* pictures of the roots grown in the container for 1 week

When a thin container packed with soil was prepared to grow the plants, neutron image provided not only water movement in soil but also morphological development of the roots, nondestructively (Fig. 7). Therefore, at first a thin Al container (3–5 mm in thickness) was prepared to analyze how the roots are absorbing water from the soil, since there was not any report about water movement within 1 mm form the surface of the roots. Then we prepared two kinds of water absorbing polymers, namely poly-vinyl alcohol polymer and poly-acrylic polymer, and compared the water supplying activity to the roots (Fig. 8). When the soybean was grown in the soil mixed with the polymers, the roots grown in poly-acrylic polymer did not grow well and changed the color, since the roots could not absorb water from the polymer. The size of the polymer did not change during the week and the color of the soil turned darker, indicating that most of the water in soil was absorbed by the plant. On the other hand, the size of the poly-vinyl alcohol polymer decreased, indicating that the water in the polymer was supplied to the roots. The study of the polymer function was further developed to apply these polymers in semiarid area to maintain water in soil of farming land.

**Fig. 9:**
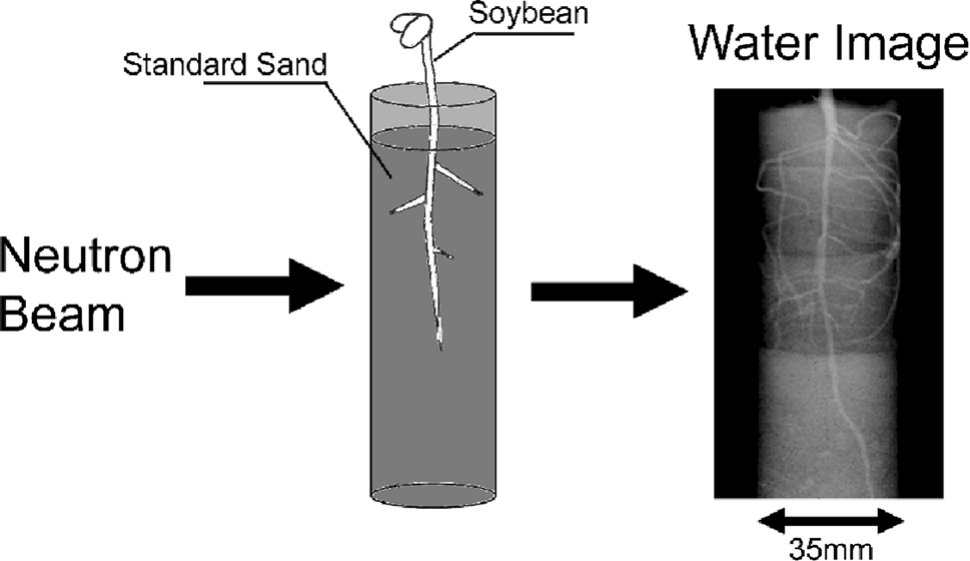
Spatial image construction of a soybean root imbedded in soil. *Note* A soybean plant grown in a pipe (3 cm in diameter) containing soil was rotated one degree by one degree till 180° and at each angle, neutron imaging was performed. Out of 180 projection images, a spatial image was constructed

**Fig. 10:**
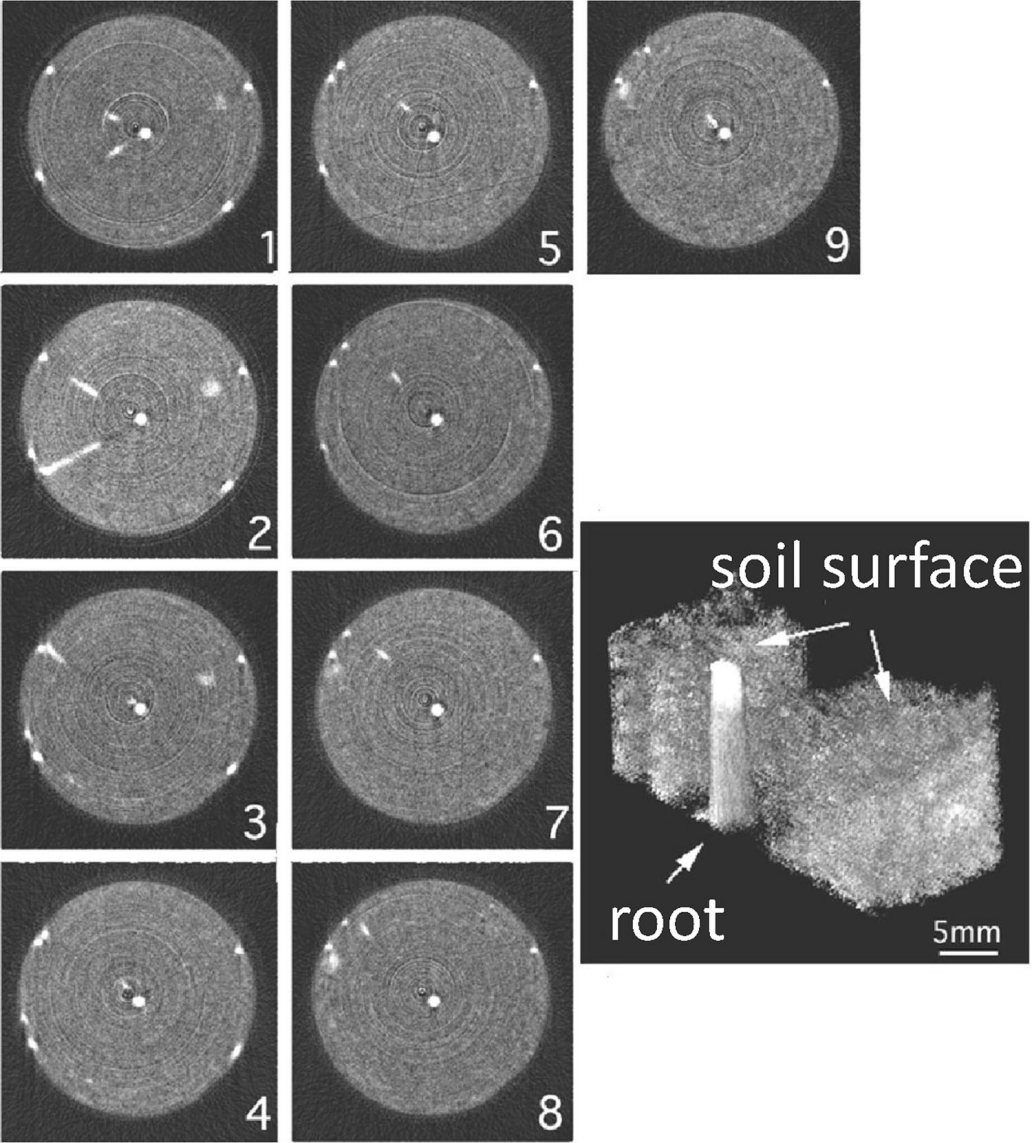
Dissection and spatial image of the soybean root imbedded in soil. *Note* Dissection images were taken every 50 μm of the height and examples of the dissection images from top to bottom (1–9) are shown. The *white dot* in the middle is the main root image and the side roots are shown to develop in radial direction and stops at the wall of the container. It was noted that in most of the images, the neighboring site of the main root is a space shown in *black*, suggesting that the root was absorbing water vapor, not water solution. *Right* image is the spatial image of the upper root and soil

The neutron imaging method was developed from two dimensional to spatial one. To get the spatial image, the plant was grown in a pipe containing soil and was rotated to get neutron images from different angles. The 180 projection images were taken while rotating the sample from 0° to 180°, and the one line image at the same height was taken out of 180 images to construct one dissection image. Then the dissection images constructed every 50 μm were piled up to produce the spatial image (Figs. 9, 10). To our great surprise, most of the adjacent place of the root in soil was an empty space (Fig. 10), which suggested that water was not absorbed from the root as solution but as vapor. There has been a long discrepancy between the soil scientist and plant physiologist for the plant growth in soil. It was suggested that the case of the water culture, which was the base of the plant physiology, might be different from that of soil culture, at least in the way, how to absorb water. The spatial image of the plant root growing in a pipe provided much information on not only water absorption movement but also the relationship between the water absorption site and side root emerging site. The application of the neutron imaging provided the way to develop the in situ physiology of the plant. Especially, the morphological development of the roots shown by neutron imaging provided the fundamental data for soil condition including the effect of fertilizer.

### Water movement using RI: water movement [28–35]

The first trial to analyze water absorption manner was studied using the cowpea plant supplied with ^18^F (half-life: 110 min) containing water, which was produced by irradiation of water with He^+^ beam. Cowpea plant was selected, since this is a draught tolerant plant and is an important crop among Asian and African countries. The plant developed a special tissue in the stem, which was suggested to function as water storage tissue but was not studied well. The water supplying function of the specific tissue under draught condition was shown using both neutron imaging and ^18^F tracer.

Then, two kinds of the cowpea plants selected in Africa were used. When water absorption manner of naturally divested cowpea plants, draught tolerant and sensitive one, were compared, it was very interesting to note that the amount of water absorbed by the draught tolerant cowpea, under normal condition, was far less than that of the sensitive one. But under draught condition, the tolerant one began to absorb much more water while the sensitive one could not absorb water. We take it as granted that the water absorption activity is very strong in draught tolerant plant so that it can survive under less amount of water in soil. Therefore, in the laboratory when we try to produce draught tolerant plant, we apt to add the water absorbing ability of the tolerant one to the sensitive one to convert the nature of the weak plant. However, naturally diverted cowpea plant showed that in usual case, water absorbing activity of the tolerant one is far behind to the sensitive one. It seems that they are saving the energy for the emergency.

**Fig. 11:**
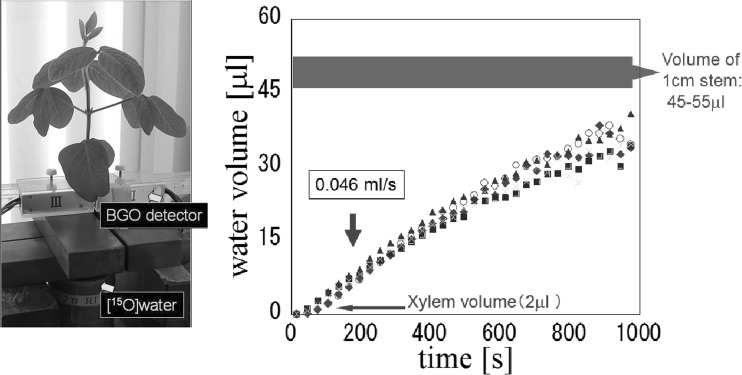
Water absorption curve of a soybean plant using ^15^O-labeled water. *Note* ^15^O-labeled water (half-life 2 min) was supplied from the root of a soybean plant. Since ^15^O is a positron emitter, the amount of water in 1 cm stem above the root was measured by a pair of BGO detectors. The measuring system was calibrated well to measure the small amount of water correctly using a phantom of the stem and harvesting the targeting 1 cm stem and measured a Ge counter. Because of extremely short half-life of ^15^O, the measurement was able to perform until about 1000 s. The half-life correction was performed to the absorption curve. The volume of 1 cm stem was 45–45 μL, and the volume of xylem tissue in 1 cm stem was 2 μL. The water absorption curve indicated that a tremendous amount of water was leaking out from xylem tissue and after about 1000 s the volume of the leaked water was close to the whole volume of the 1 cm stem

**Fig. 12:**
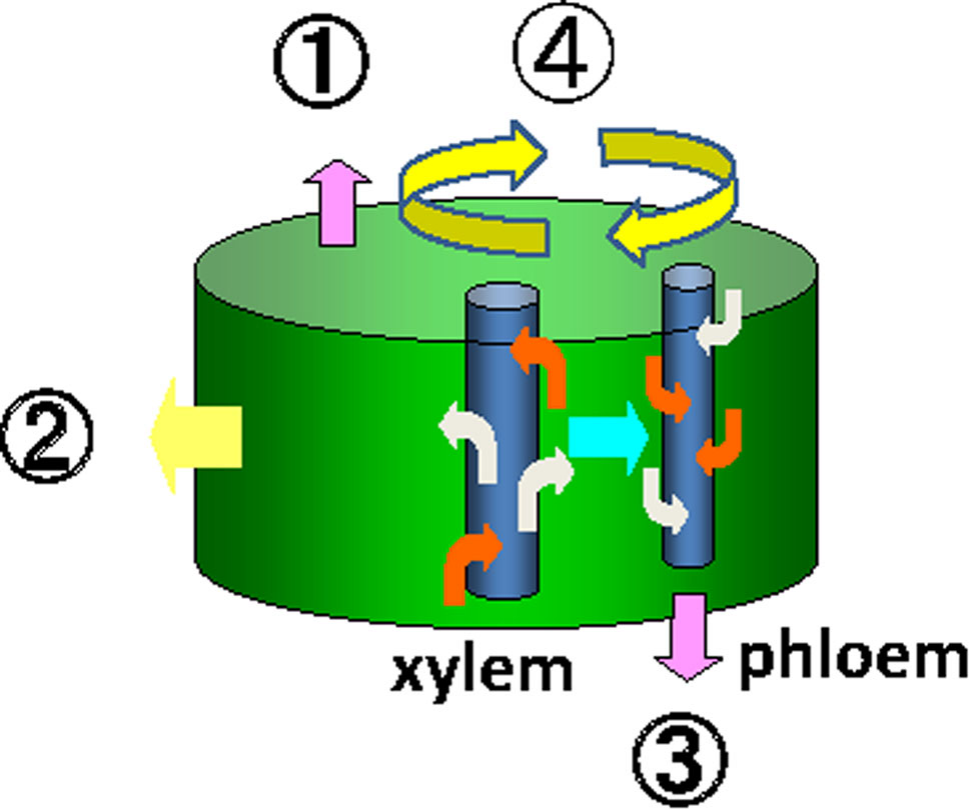
The route of the water after leaking out from xylem tissue. *Note* There are four routes for the water to leak out from xylem tissue, namely go upward through the tissue other than xylem, lose from the surface of the stem, go downward through the connected part from xylem to phloem, and return to the xylem tissue and go upward. All the possibilities of the route were investigated and the route was found to be the last one returning to the xylem, which is also evident from Fig. 13

Applying ^18^F-labeled water, water storage function of the tissue in the draught resistant cowpea plant was demonstrated. However, the different movement of ^18^F in water from that of ^15^O-labeled water was shown. Therefore, ^15^O-labeled water (half-life: 2 min) was used to measure trace amount of water movement. The measuring system was prepared and the performance of the system was developed so that the trace amount of the water moving in the soybean could be measured. Since ^15^O is a positron emitter, a pair of BGO detector was used and their performance was studied. Using the system, circulation of water movement within the stem was found for the first time. That is, tremendous amount of water was leaking out horizontally form the xylem tissue, which has been regarded as a mere pipe to transport water from root to the up-ground part (Fig. 11). After examining every possibility of the route for the water flowing out from the xylem, it was found that the leaked water from the xylem entered the xylem tissue again and then transferred to the upper part (Fig. 12). Because of the constant returning manner of the leaked water to the xylem, the water velocity in the xylem tissue was kept constant. The constant flow rate of the water in xylem tissue was measured by preparing three pairs of BGO detectors. From the known distance between the detectors and from the time when they first detected the radiation from ^15^O-labeled water coming up from the root, the flow rate between the detectors was found to be constant.

**Fig. 13:**
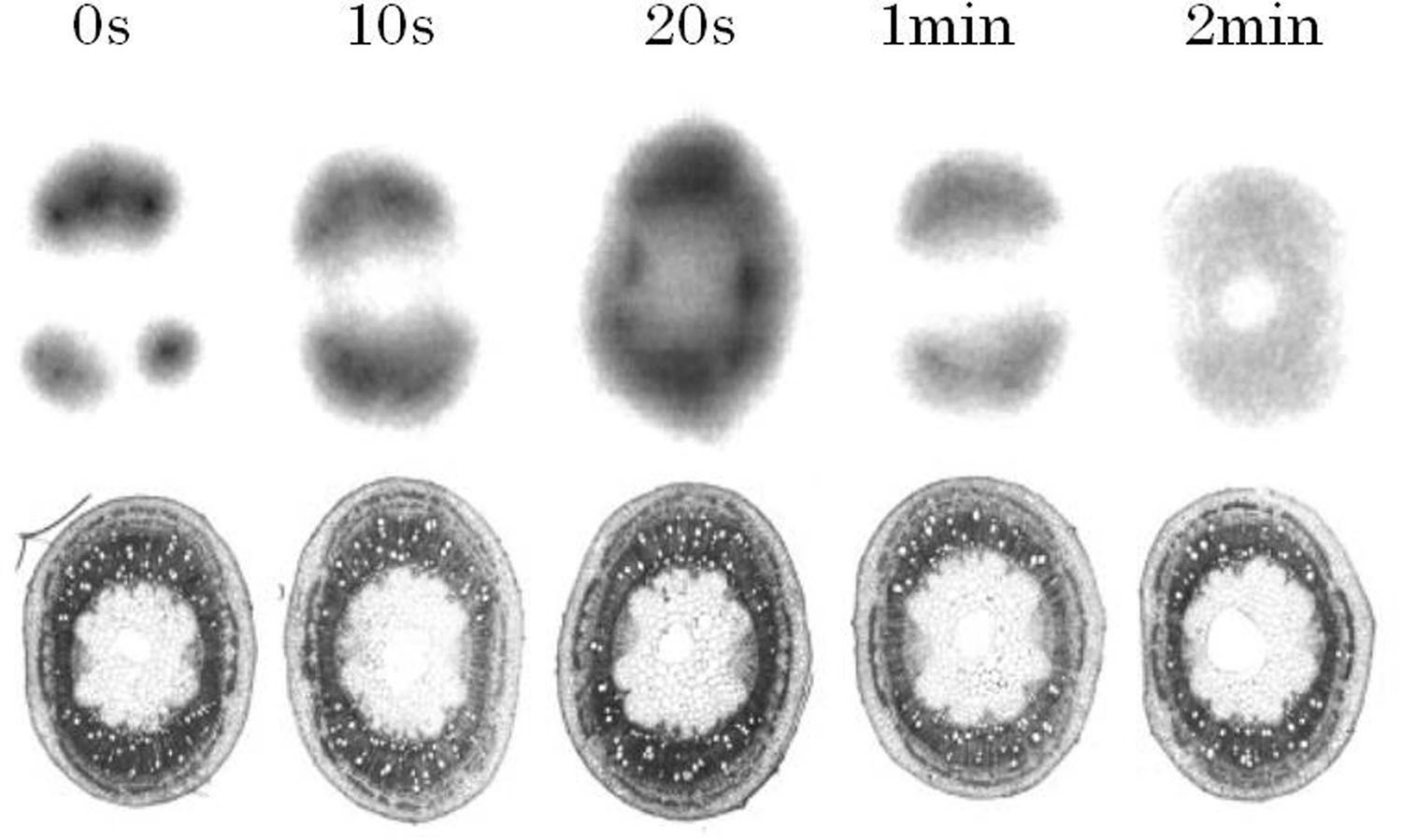
Water leakage imaging using ^3^H-labeled water. The beta-ray energy emitted from ^3^H was too low to detect from outside the stem; the stem had to be harvested each time for imaging; therefore all samples were derived from different plants

This water movement was also confirmed using ^3^H-labeled water (Fig. 13). In the case of ^3^H, the beta-ray energy from ^3^H was too low to be detected from outside the plant. Therefore, ^3^H-water was supplied for 5 s and the stem was cut after, 0, 10 and 20 s, 1 and 2 min and autoradiography was taken by an imaging plate. As is shown in the figure, the flowing out water from xylem tissue was spread out to all of the stem tissue after 20 s and gradually returning to the xylem. Through simulation, the newly absorbed water from the root replaced half of the water already existed in the stem within 20 min. This water circulation movement was also found even under high humidity when the transpiration, water loss from leaves, was hardly expected.

### Neutron activation analysis (NAA): inorganic element distribution [36–60]

**Fig. 14:**
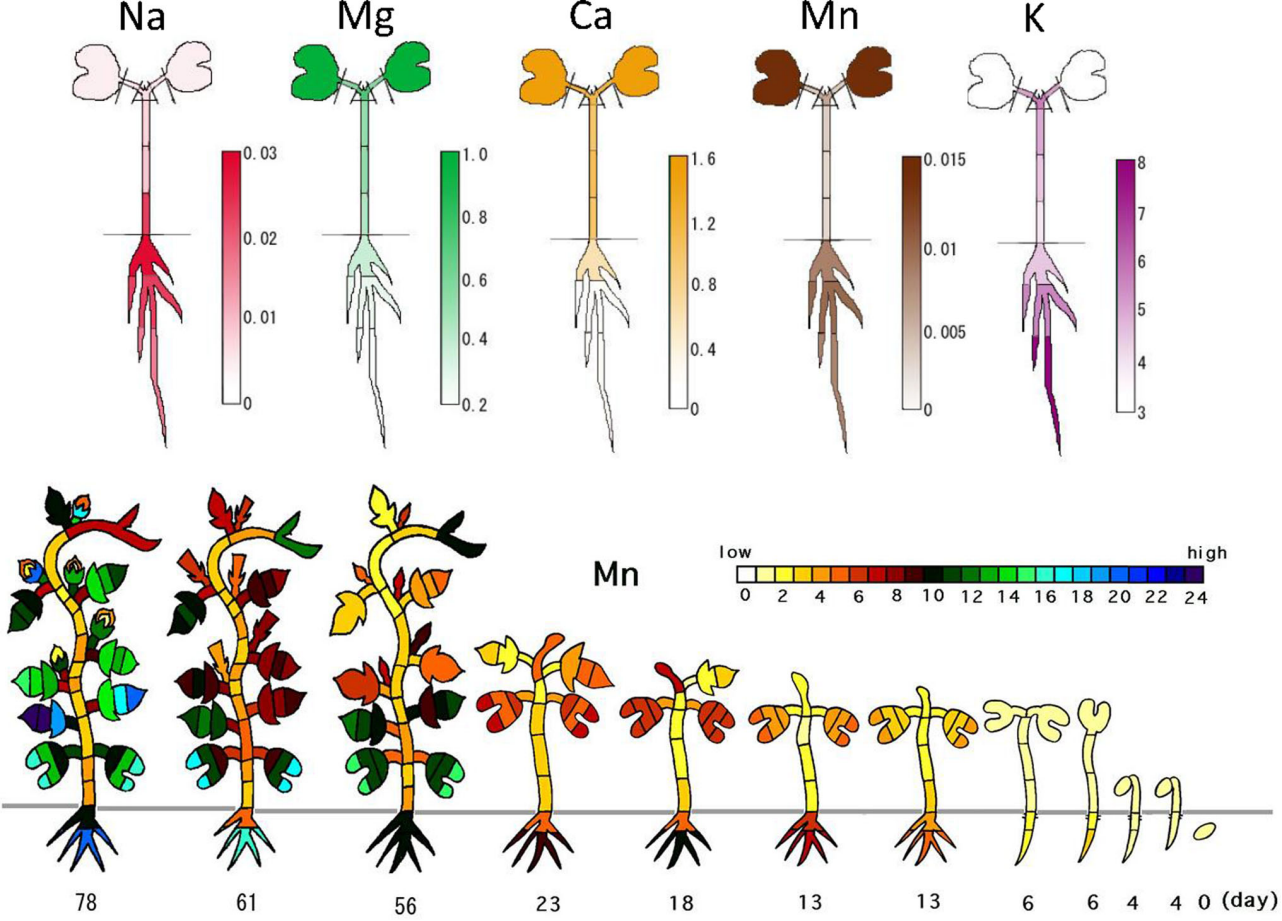
Neutron activation analysis of morning-glory (1). *Note* Tissues of morning-glory was separated and NAA was performed. *Top* 1 week seedling; *bottom* from germination to seed formation. The pseudo-color was employed according to the concentration of the element. About ten elements were measured in each tissue but only representative elements were are shown

**Fig. 15:**
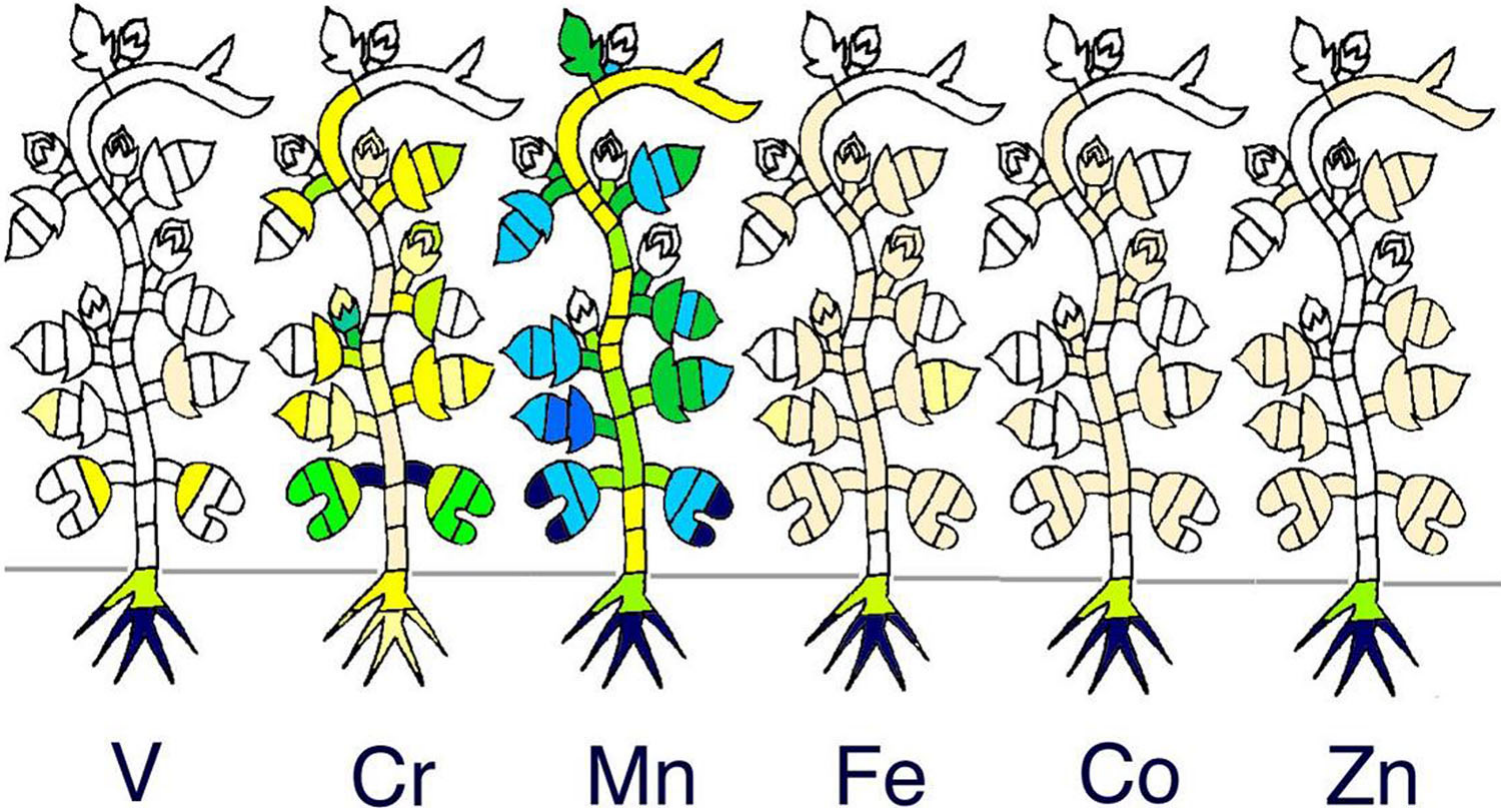
Neutron activation analysis of morning-glory (2). *Note* The heavy element distribution after 78 day of germination is shown. Heavy elements tend to accumulate in roots except for Mn and Cr. Pseudo-color was employed according to the concentration of the element

NAA was performed for many kinds of plant tissues. An element specific distribution profile was found in barley leaves. Each leaf showed gradient of the elemental concentration and the profile hardly changed throughout the developmental stage. Similarly, when morning-glory was analyzed from germination to seed formation by NAA, specific distribution pattern was found for each inorganic element and the tendency was confirmed in a 7-day seedling (Fig. 14). The result showed that K concentration in the leaf stem was always high and meristems are free from toxic elements. In the case of Cl and Br, their concentrations in the plant decreased during the developmental stage, suggesting that these elements evaporated and lost from the plant. Most of the heavy elements tended to accumulate in roots except for Cr and Mn (Fig. 15), suggesting that the element which has many chemical valences can easily move. Cobalt concentration in stem was higher compared to those of the other tissues in above-ground parts of the plant. Although the concentration of the Co is important to grow excellent grass in meadows, the reason is still not clear.

**Fig. 16:**
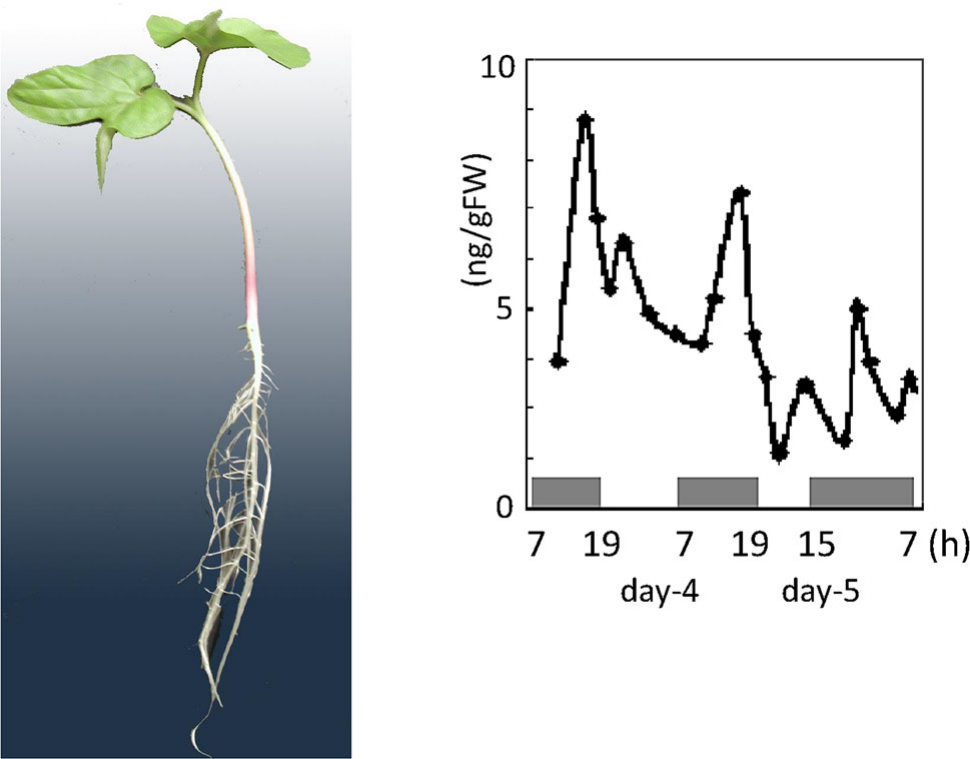
Al concentration in root tip by activation analysis. The Al concentration in the root tip of the morning-glory was measured after 4 and 5 days of germination. The light period was from 7:00 to 19:00 during the day but on day 5, daytime was reduced and dark period began at 15:00. The overall Al concentration decreased in the root tip, at the same time a fluctuation of the Al concentration was observed. At the end of the dark period, Al concentration increased and then decreased, suggesting the secretion and absorption of Al in the root tip

Sensitivity of Al in NAA is extremely high making it easy to detect in any sample. The Al concentration in root tip was measured in a morning-glory plant after 4 and 5 days of germination. The light period lasted during 07:00–19:00 for 4 days but on day 5 it ended at 15:00. There was a high fluctuation of Al concentration in root tip and was highest just before the end of the dark period, though the total amount of Al decreased gradually (Fig. 16). The fluctuation of Al indicates absorption and secretion of the Al from roots. Though the absorption and secretion of other elements such as K are known, Al has not been studied well since there are not any suitable and easily available radioisotopes for Al.

Using a large set of data on elemental profile of a whole plant, attempts were made to construct an element recycling scenario in soil–plant system since it is known that more than half of the N contained in a leaf was recovered by the plant before falling off to the ground. Before ^28^Mg tracer was available, Mg content in plant tissue was analyzed mainly by NAA. It was found that the amount of Mg absorption increased during the day time and drastic change in Mg concentration at the center cells of meristem appeared when flowering was induced; these results prompted further study to prepare ^28^Mg tracer.

NAA as well as prompt gamma-ray analysis was applied to food samples too, such as onions and beefs produced at different places, to measure levels of as many elements as possible. The elemental profile of the food showed specific features of elemental ratios, representing the production sites of the vegetables and cows.

### Development of radioisotope imaging (RI) systems [61–80]

Two types of the real-time RI imaging system, namely macroscopic and microscopic, were developed. Both systems are designed to use conventional RI so that it is possible to perform imaging in our laboratory and not at special laboratory with special facilities. Although it is possible to produce RIs by an accelerator and use the RI at special facilities, the frequency of doing experiment is limited and the environment for plant research including growth chamber has to be installed. Therefore, it was much preferable and efficient to develop the imaging system if we could use the conventionally available RI. Plant sample has another problem: the up-ground part requires light and the root does not. To perform imaging from root in dark to up-ground part in light, fluorescent imaging method cannot be employed since it requires dark condition only. It was our first trial to develop real-time imaging system using conventional RI.

#### Macroscopic real-time RI imaging

**Fig. 17:**
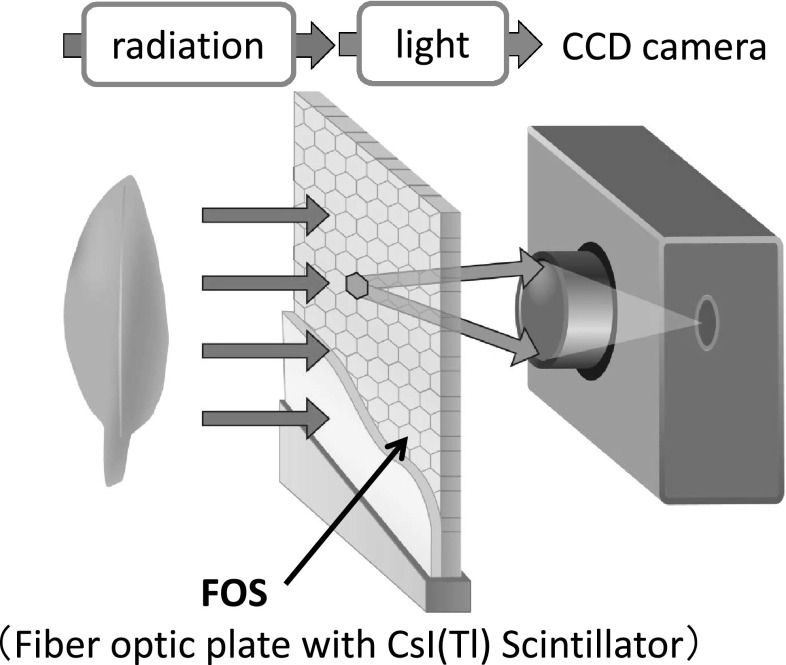
The principle of real-time RI imaging system. RI was supplied to the plant, the emitted radiation was converted to weak light by the Cs(Tl)I scintillator deposited on the surface of the fiber optic plate (FOS). Then the light was collected by a highly sensitive CCD camera

The principle of RI imaging is shown in Fig. 17. Radioisotopes were supplied to the plant sample and the radiation emitted from the plant was converted to weak light by Cs(Tl)I scintillator deposited on a fiber optic plate (FOS). The light image was then collected by a highly sensitive CCD camera and processed through computer. The kind and thickness of the scintillator was studied and 100 μm deposition of Cs(Tl)I on FOS surface and was found to be suitable for this study. The sensitivity was more than ten times higher than that of an imaging plate (IP) which is now widely used for autoradiography. The higher sensitivity of the imaging means the accumulation time to get imaging is short, indicating that the successive images can be recorded smoothly and made to a movie.

**Fig. 18:**
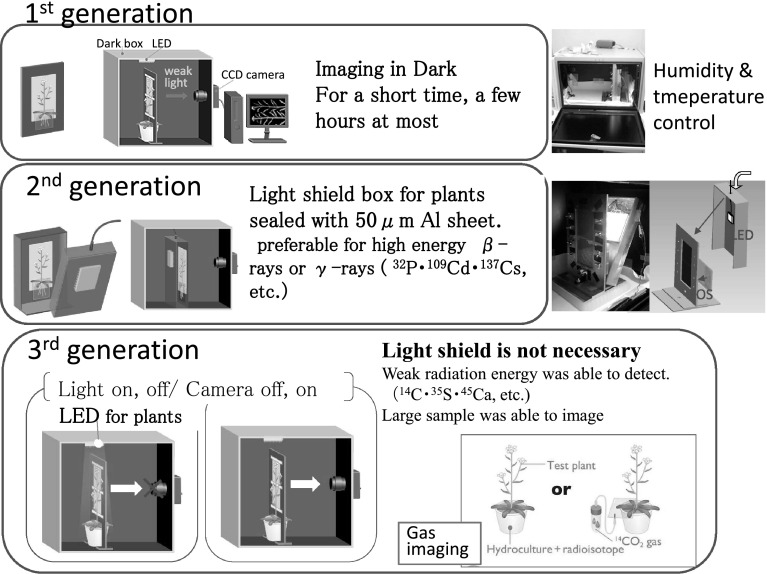
Development of the real-time RI imaging system. *Note* Since the CCD camera employed to image the light from the scintillator is highly sensitive to light, everything was kept in dark in 1st generation to protect the camera. Then the light shielded plant box was prepared and light could be irradiated to the up-ground part of the plant in the 2nd generation. In the 3rd generation light was off when imaging was performed so that weak radiation energy could be detected

The 3rd generation (Fig. 18) macroscopic imaging system has now been developed. Everything had to be kept in dark in the 1st generation; then the plant box was introduced in the 2nd generation where only the upper part of the plant was irradiated with light. Finally, the light was off only when camera was on in the 3rd generation so that weak radiation energy from radioisotopes such as ^14^C, ^35^S and ^45^Ca, etc. could be easily detected. With the 3rd generation system, it is possible to image relatively large plants which cannot grow in a plant box. Now we are able to image how CO_2_ gas is fixed by the plant by introducing ^14^CO_2_ gas to the plant.

**Fig. 19:**
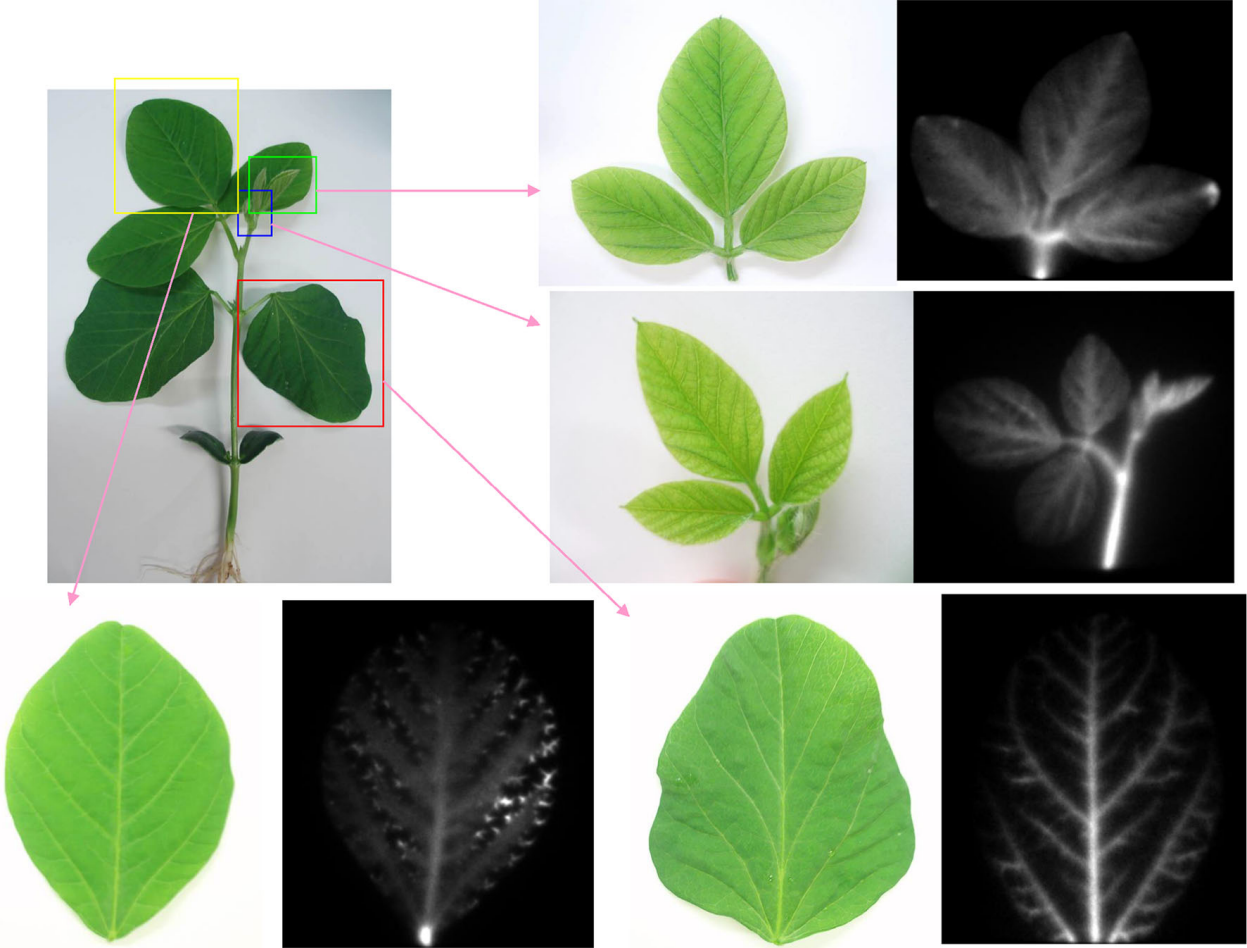
Real-time imaging of ^32^P-phosphate taken up in a soybean plant. *Note* ^32^P-phosphate was supplied from the root and the accumulation pattern of ^32^P was recorded. ^32^P-phosphate first moved up to the youngest tissue and then to the relatively older tissue. Among the leaves, ^32^P was found to be highly accumulated between the vain shown as *dots* (*bottom left* image)

**Fig. 20:**
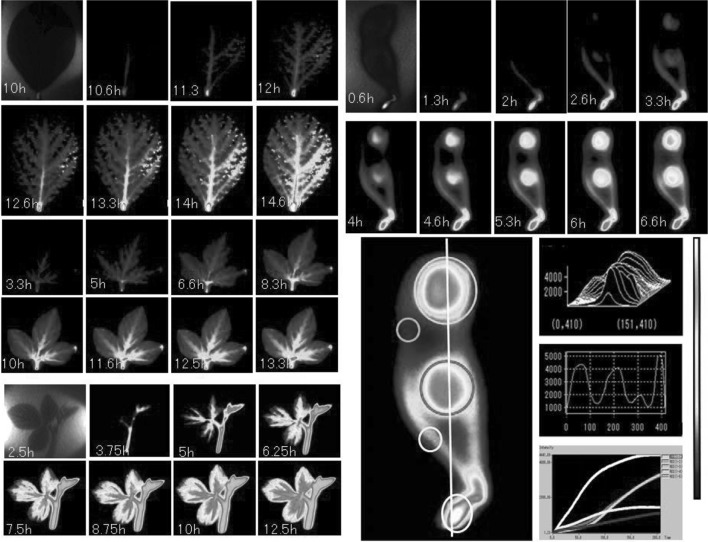
Image analysis of the ^32^P-phosphate movement in plant tissue. The lap-time images shown in Fig. 19 were recolored based on the counts in the pixels. While analyzing ^32^P accumulation in seeds in the pod, no difference was found in the transferring speed, or in the amounts of the two seeds (*bottom right*)

**Fig. 21:**
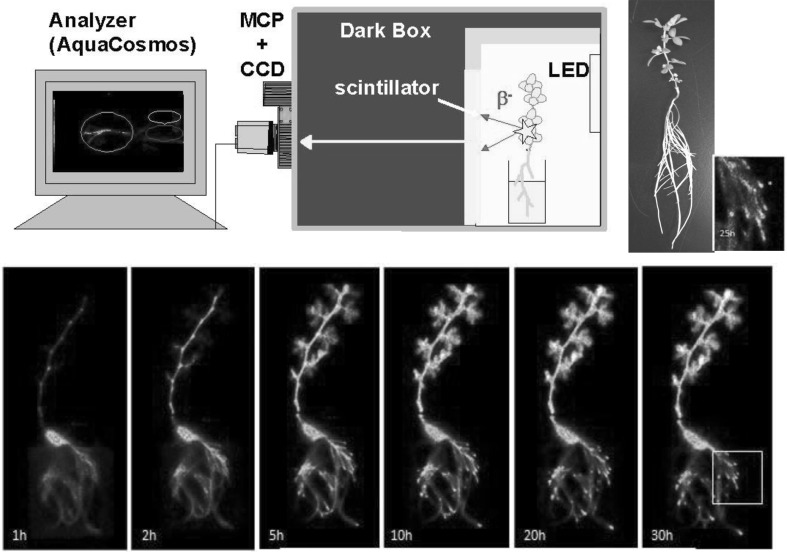
Real-time imaging of ^32^P-phosphate taken up in a Birdsfoot trefoil plant. *Note* ^32^P-phosphate was supplied to a Birdsfoot trefoil plant in a plant box and successive images of ^32^P-phosphate movement were taken. Pseudo-color was employed to show the amount of ^32^P in the image

The first trial was to image ^32^P-phosphate uptake movement in a soybean plant using the 1st generation system, since phosphate is reported to move fast in a plant and it sometimes represent the movement of water. When phosphate was supplied, it was transferred fast to the youngest leaves and then gradually transferred to the older tissue. It was interesting that the accumulation pattern of ^32^P-phosphate was different among the leaves and there was high accumulation of phosphate between the vain, shown as dots, in one kind of the leaf (Fig. 19). Since the image is based on radiation counts, we could treat the image numerically. For example how ^32^P was accumulated in seeds in pod was analyzed, and found that there was no difference of the transferring speed as well as the phosphate delivery between the two seeds (Fig. 20 bottom right). The lap-time images shown in Fig. 20 was treated with pseudo color. From the radiation image, we could not know the chemical form of the ^32^P. Therefore, we performed a chemical separation of the phosphate in the tissue and found that the chemical form of ^32^P was maintained as phosphate at least during the first 30 min after the treatment. Birdsfoot trefoil or Arabidopsis were used to image ^32^P-phosphate movement, which led to the phosphate transporter research in root tips using both macroscopic and microscopic imaging system (Fig. 21).

**Fig. 22:**
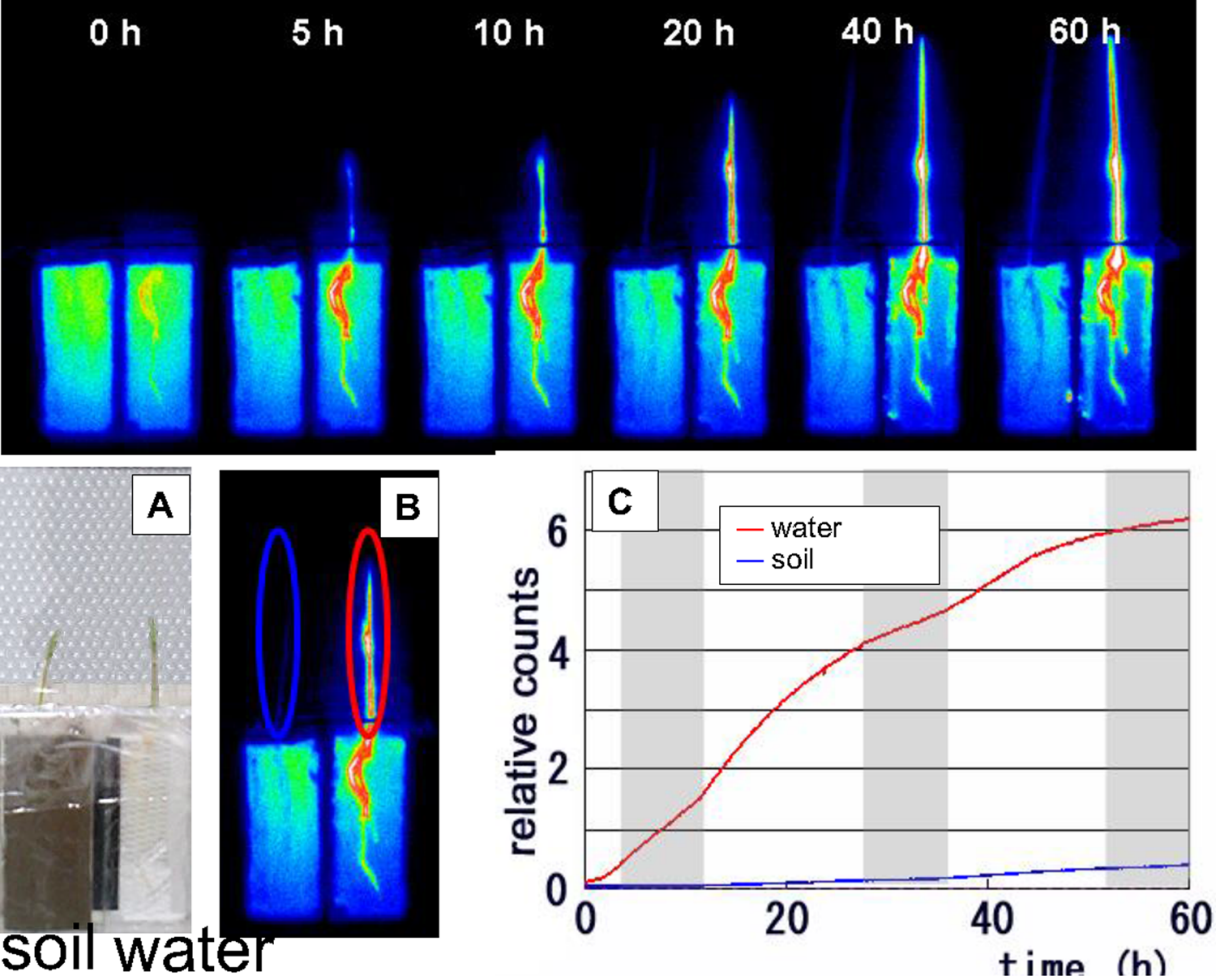
Real-time imaging of ^32^P-phosphate taken up in a rice plant grown in soil and water. A rice plant was used to compare the ^32^P-phosphate absorption in soil and in water culture. Since phosphate was adsorbed to the soil, rice roots eluted the phosphate from the soil and then absorbed the ^32^P-phosphate while growing which was shown by the increasing shade of the root in the soil. Therefore, the ^32^P-phosphate uptake was very low compared to that in water culture and grows slowly. Whereas in water culture, the amount of the ^32^P-phosphate absorbed to the plant was more than ten times higher than that in soil culture. But generally, the yield, amount of seed, in water culture is much lower than in soil culture

In order to show the difference between water and soil cultures, ^32^P-phosphate was supplied to rice seedlings and the movement of ^32^P was traced for 60 h. The uptake amount of ^32^P-phosphate was more than ten times higher for the plants grown in water culture compared to that in the soil culture. The growth of the plant is much faster when grown in water culture. Phosphate is known to adsorb on to the soil and plant root was able to take up the phosphate from the soil in the vicinity of the root which is shown as remaining root shape in the soil (Fig. 22). However, the yield of the rice grown in water culture is low compared to that grown in soil, which might suggest that when the nutrient was supplied in ion form it was easy to absorb and the plants become rather inactive in generating the next generation under too comfortable condition.

#### Tracer production (^28^Mg, ^42^K) [81–98]

**Fig. 23:**
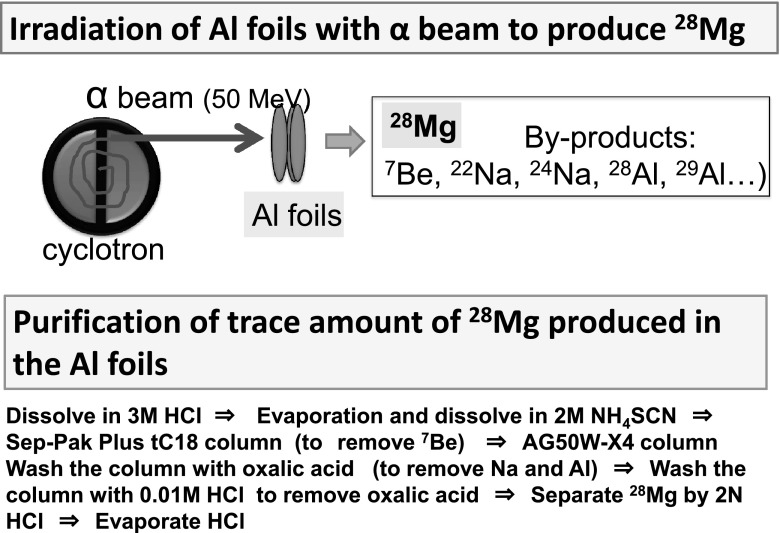
Production of ^28^Mg for tracer use. *Note* Ten sheets of an Al foil (1 cm × 1 cm) were irradiated with He^+^ beam to produce ^28^Mg (half-life: 21 h). After purification of ^28^Mg from the target by eliminating by-products, such as ^7^Be, ^24^Na etc., ^28^Mg was used as a radioactive tracer for imaging and tracer work

^28^Mg (half-life: 21 h) and ^42^K (half-life: 12 h) were produced and applied for the first time in plant study. Aluminum film target was irradiated with He^+^ beam and ^28^Mg produced in the target was radiochemically separated (Fig. 23). Magnesium is a very rare element among the other inorganic elements, keeping constant concentration in the plant species, suggesting that the role of Mg is to keep the homeostasis of the plant activity. Sometimes it has been reported that the behavior of Mg is similar to that of Ca because of they belong to the same group in the periodic table. However, because of the overwhelming amount of Ca compared to that of Mg in plants, popular fluorescence staining method could not distinguish Mg from Ca for any meaningful conclusion. Using ^28^Mg tracer, basic properties of Mg movement in plants were clarified and the trial to find Mg specific transporter is now being developed.

**Fig. 24:**
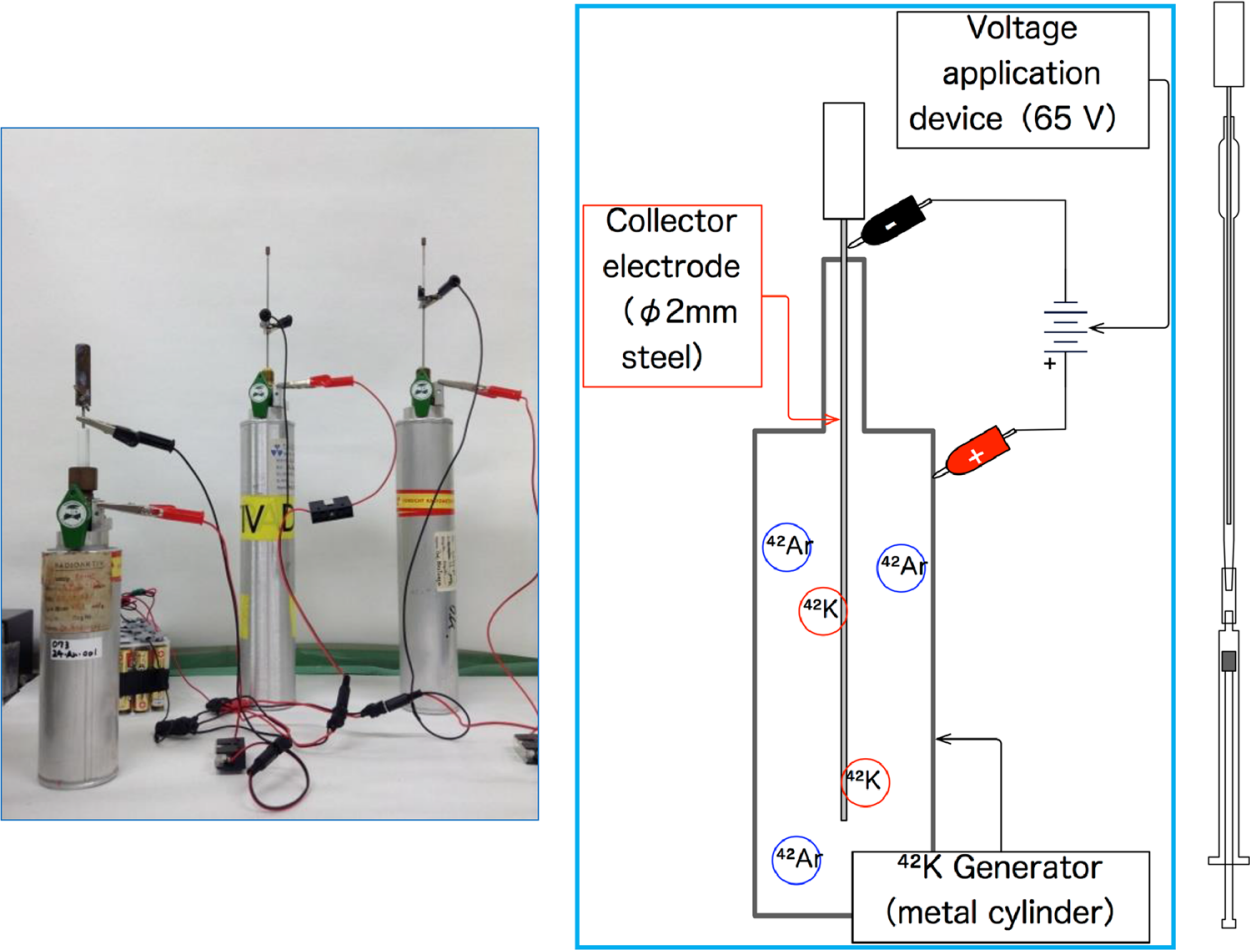
Preparation of ^42^K from ^42^Ar. *Note* ^42^K (half-life: 12 h) was prepared from ^42^K generator, where ^42^Ar (half-life: 30 a) gas was sealed in a cylinder. The electrode was inserted in the cylinder and 65 V was applied. After 3–4 days, the electrode was taken out and washed in water solution where ^42^K, decay product of ^42^Ar, collected to the electrode was dissolved as a carrier free ^42^K^+^ ion

^42^K is another nuclide applied to plant study for the first time. It was prepared from ^42^Ar (half-life: 30 a) gas by a milking method (Fig. 24). Potassium study was rather behind in plant research because of the lack of suitable tracers. Instead of K tracers, ^86^Rb was used to trace the K movement but there was a difference in movement. When all the K in the plant was replaced with Rb, the plants could not grow further. ^42^K tracer was useful, after Fukushima Daiichi nuclear accident, to study the relationship between the behavior of K and Cs, such as to analyze the reduction mechanism of ^137^Cs absorption by applying K to the plants.

#### Imaging the movement of various RI tracers including ^14^CO_2_ gas

**Fig. 25:**
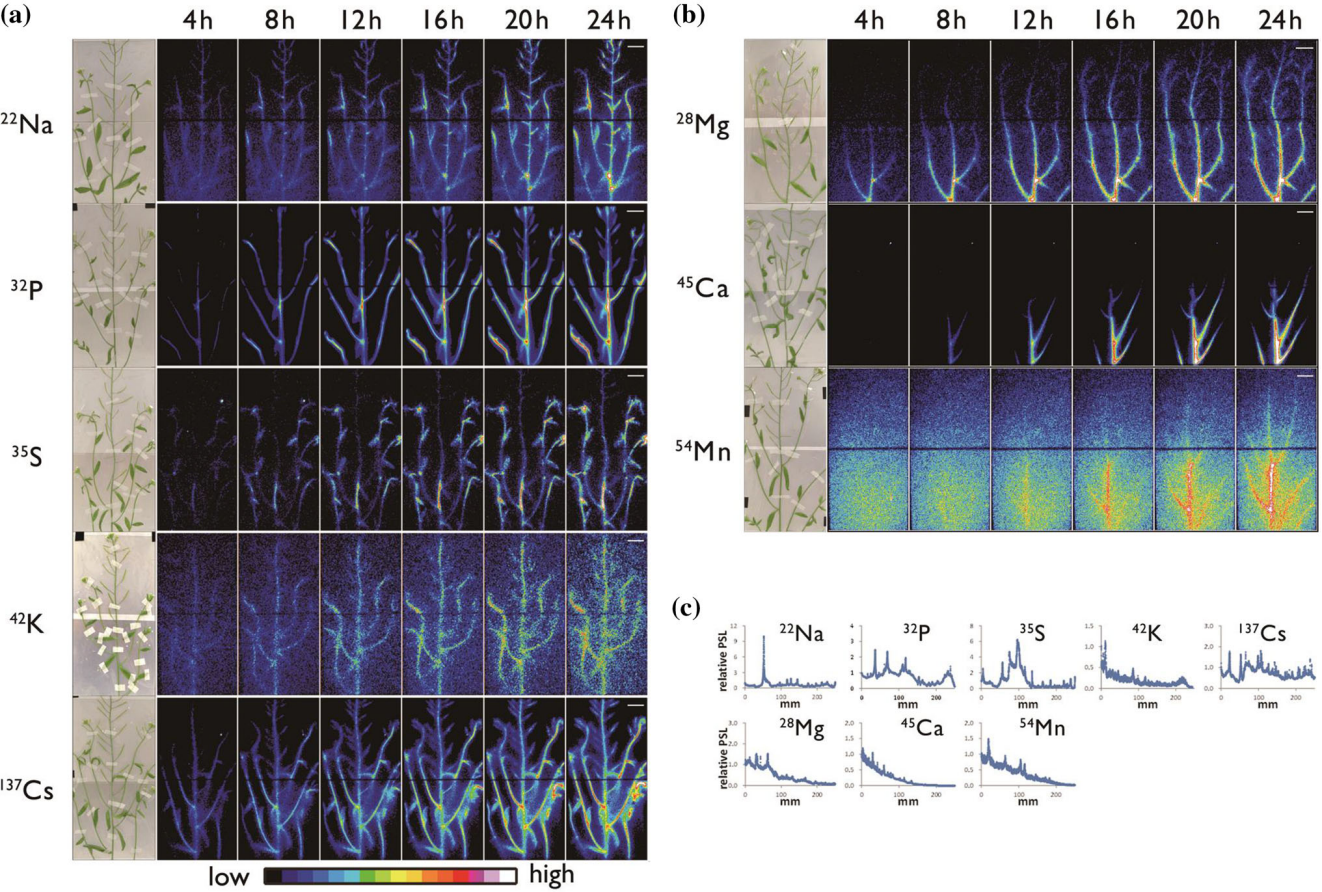
Real-time imaging of eight inorganic elements in an Arabidopsis plant. *Note* The absorption and distribution images of radioisotopes of eight inorganic elements, namely ^22^Na, ^28^Mg, ^32^P, ^35^S, ^42^K, ^45^Ca, ^54^Mn, ^137^Cs, in the up-ground part of Arabidopsis during 24 h are shown. Pseudo-color was employed

As mentioned earlier, some of the radioactive nuclides applied to our systems were: ^14^C, ^22^Na, ^28^Mg, ^32^P, ^33^P, ^35^S, ^42^K, ^45^Ca, ^54^Mn, ^55^ Fe, ^59^Fe, ^65^Zn, ^86^Rb, ^109^Cd, ^137^Cs, etc. In the case of macroscopic system, tracer movement from root to up-ground part of Arabidopsis was visualized using various radioactive nuclides. Figure 25 shows how the inorganic elements, namely ^22^Na, ^28^Mg, ^32^P, ^35^S, ^42^K, ^45^Ca, ^54^Mn, and ^137^Cs, absorbed from roots were transferred to the up-ground part of Arabidopsis within 24 h. Among the nuclides, ^28^Mg, ^45^Ca, and ^54^Mn were very slow to move up-ground part from the root.

**Fig. 26:**
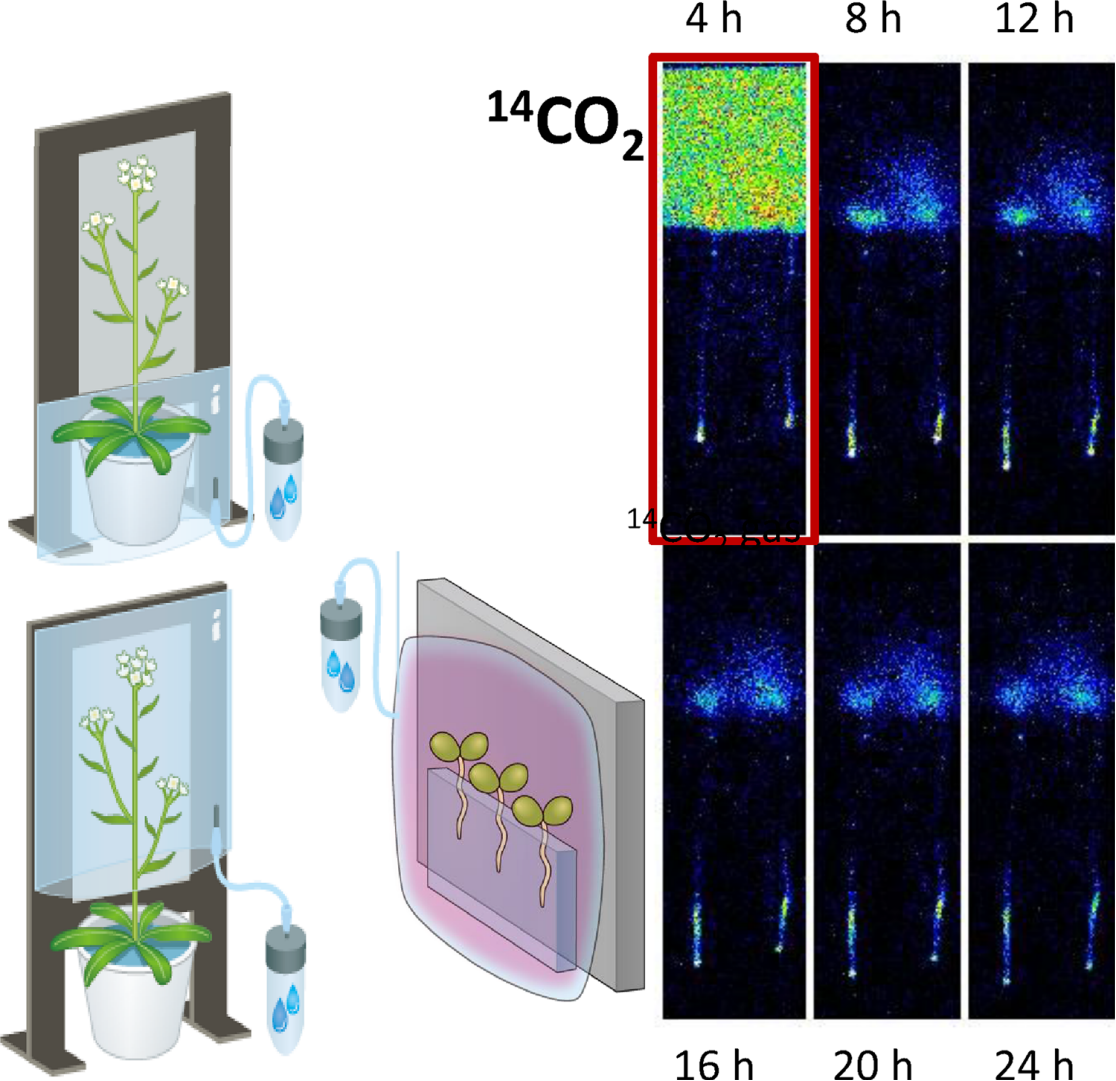
Real-time imaging of ^14^CO_2_ gas fixation. *Note* ^14^CO_2_ gas was generated by the addition of lactic acid to NaH^14^CO_3_. Then the ^14^CO_2_ gas was supplied to the rosette or other part of the up-ground tissue of the Arabidopsis to investigate whether there is a preference of the transfer route between the photosynthate produced in different tissue (*left*). ^14^CO_2_ gas was supplied for 15 min from the leaf of the seedling and the accumulation of photosynthate is shown (*right*)

We were even able to image ^14^CO_2_ gas fixation manner. Figure 26 shows the ^14^CO_2_ gas application from different sites of the plant. When ^14^CO_2_ gas was supplied to the rosette of the Arabidopsis, the photosynthate was moved swiftly to the meristem of the main stem and roots, whereas when applied from the other part of the plant, the photosynthate moved to the meristem of the branch stem only and was not transferred to the roots. What derives the different route of the photosynthate is not presently known.

**Fig. 27:**
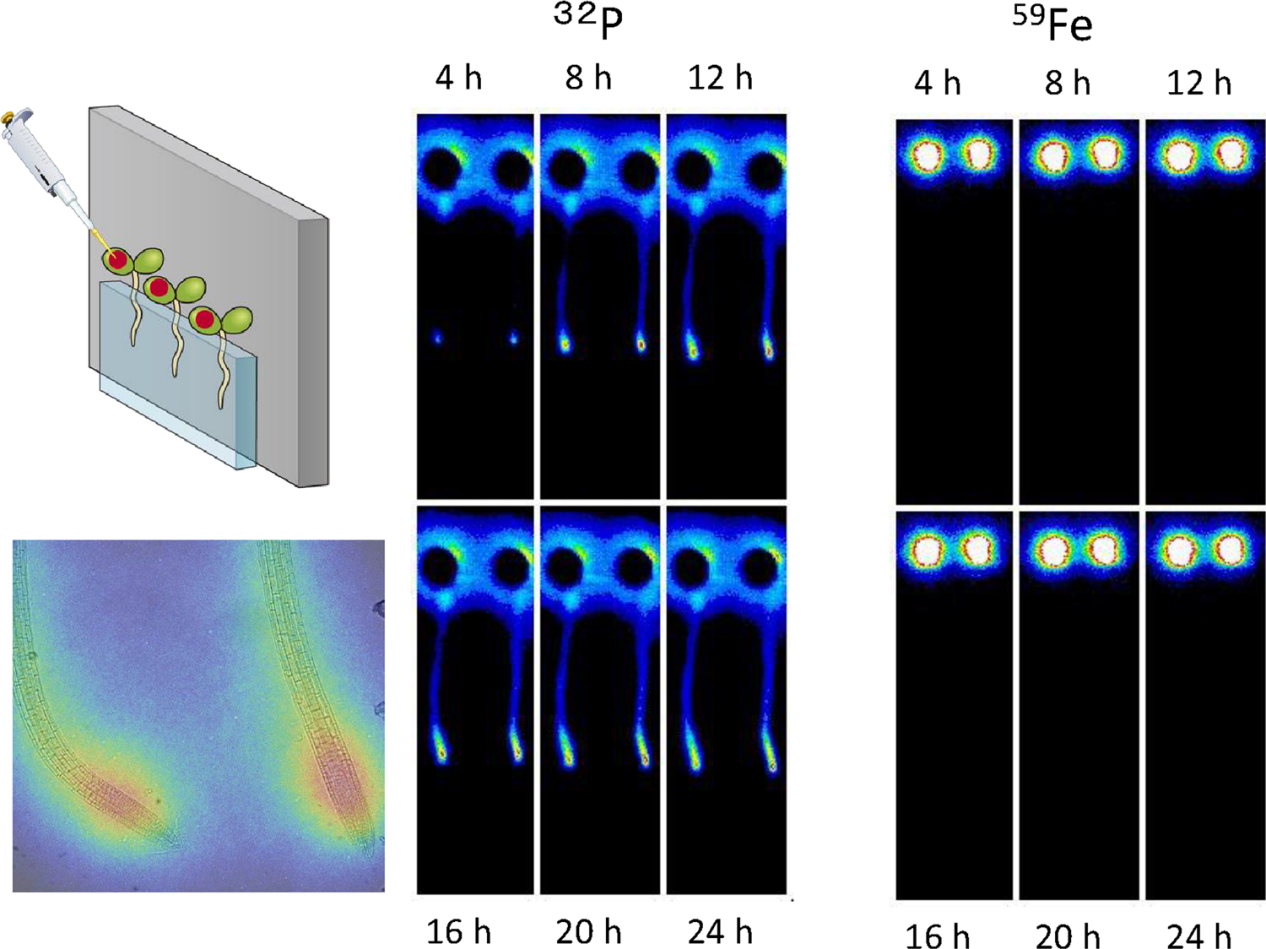
Real-time imaging of inorganic elements from root. Downward movement of the inorganic elements in Arabidopsis was visualized. When applied to the leaf of the seedling, downward movement of ^32^P was fast and was accumulated in the root tip. The root tip image accumulating ^32^P is shown at the *bottom left* figure. In the case of ^59^Fe, downward movement was not observed during the first 24 h and a large fraction of it remained in the leaf

The 4-h application of ^14^CO_2_ gas to the up-ground part of the Arabidopsis seedling showed that the photosythate went downward and accumulated to the certain part of the root tip, indicating that the photosythate produced from this 4-h application of ^14^CO_2_ gas actually produced certain part of the root tissue. Besides ^14^CO_2_ gas, downward movements of the inorganic elements were observed. Among the elements, ^32^P or ^35^S moved toward the root tip when supplied from the leaf, whereas most of ^59^Fe or ^45^Ca stayed on the leaf. Figure 27 shows the movement of the two representative nuclides, namely ^32^P and ^59^Fe supplied from the leaf during 24 h with the microscopic image of ^32^P in root tips.

#### Microscopic real-time RI imaging system

**Fig. 28:**
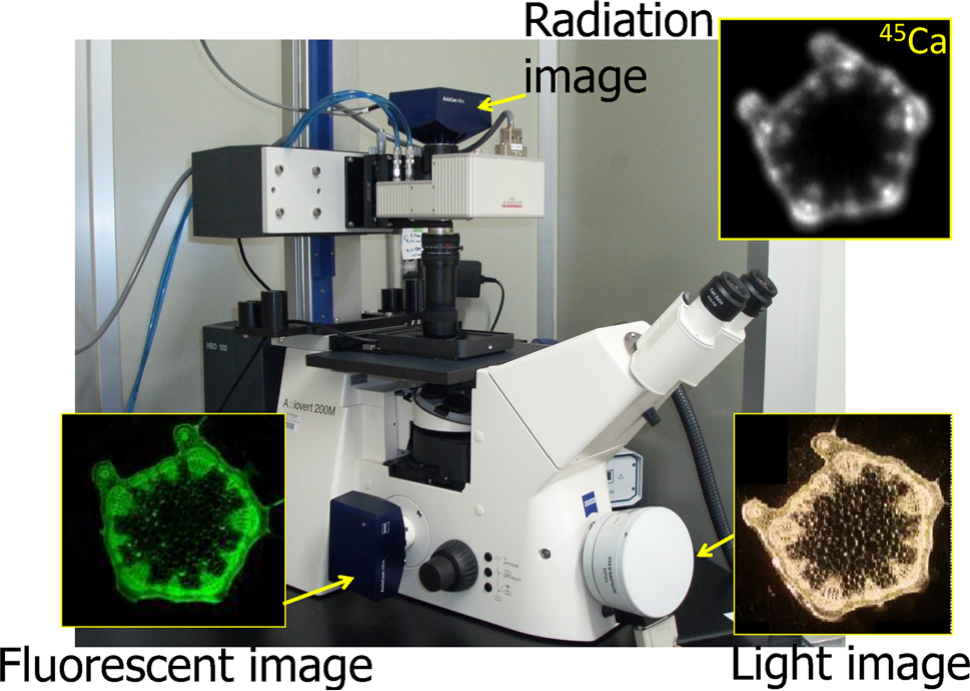
Microscopic imaging system. *Note* Light image, fluorescent image and radiation image of Ca in the stem of a soybean plant are shown

**Fig. 29:**
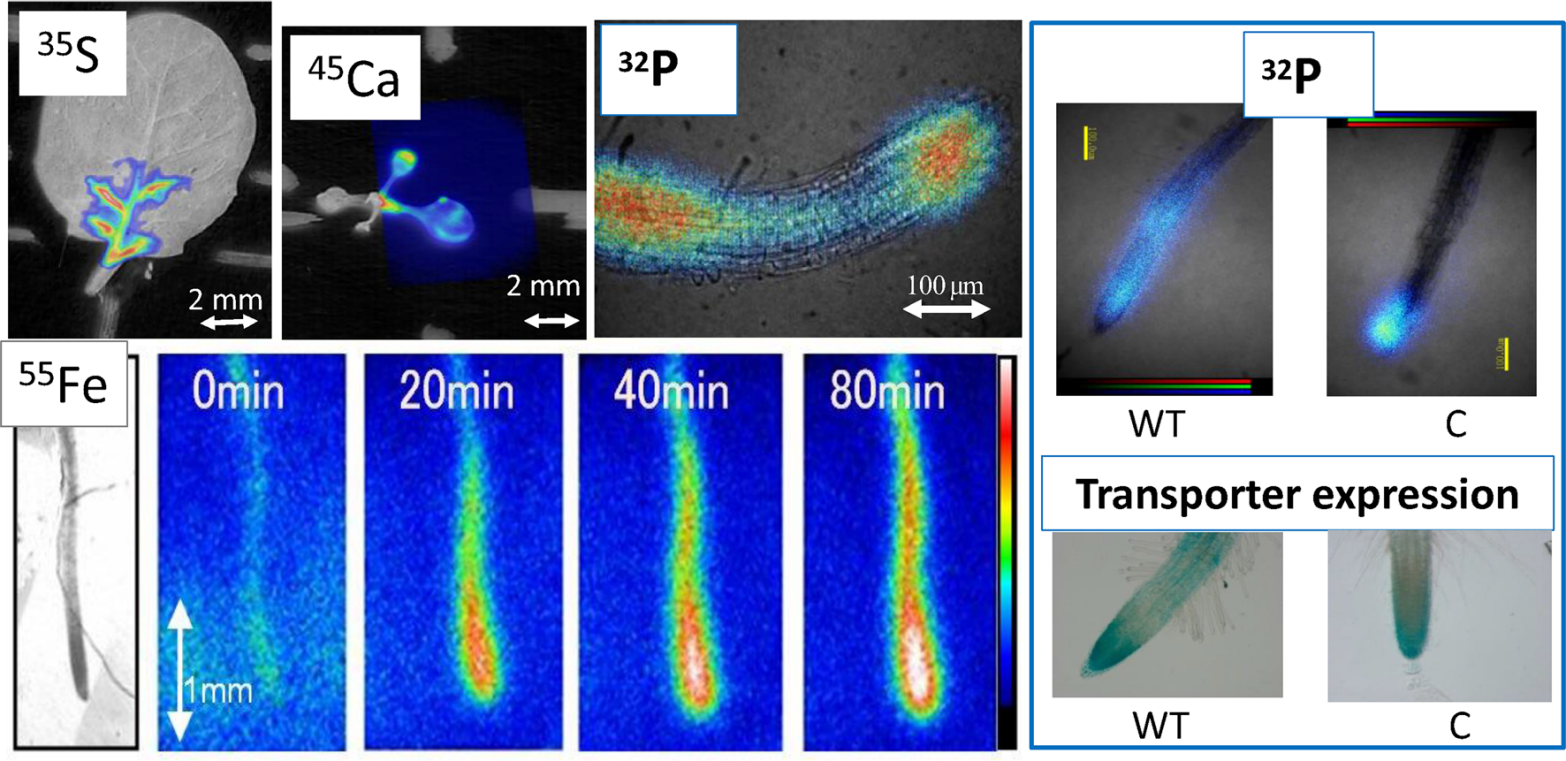
Real-time microscopic RI image. *Note* RI images taken by the microscopic imaging system are presented. Arabidopsis was employed in all cases. ^35^S and ^45^Ca images are the in leaves when they are transferred from roots. ^55^Fe was supplied to the root and the accumulation images were taken successively. ^32^P distribution in the root in the middle was the superposed image of ^32^P to the light image. *Right figure* shows how ^32^P are distributed in wild type (WT) and in a mutant (C). The phosphate transporter gene expression of the mutant root tip, shown at the *bottom*, was found to be the same as that of the ^32^P distribution

The microscopic system was prepared by modifying a fluorescence microscope so that three kinds of images, namely light image, radiation image and fluorescence image, could be obtained at the same time (Fig. 28). The representative images of Arabidopsis applying ^32^P, ^35^S, ^45^Ca and ^59^Fe are shown in Fig. 29. In the case of Arabidopsis root, ^32^P movement even in a fine root of diameter of about 100 μm was analyzed.

There were many mutants created in Arabidopsis plants which limit the expression site of the transporter gene, such as phosphate. A mutant (C) was produced to express the transporter gene only at root tip. The gene expression of the mutant (C) was shown by fluorescent staining method. But at the same time, it is important to know whether phosphate is also transported at the same site or not. Therefore, ^32^P-phosphate image was taken for the mutant (C), and was shown that the site where transporter was expressed was also the site transporting the ^32^P-phosphate (Fig. 29). Though the expression of element specific transporter gene is gathering attention, gene expression shows only the gene activity and does not necessarily mean the element movement itself; therefore radioactive tracer imaging could provide the direct evidence of gene function.

### Fukushima accident related work [99–133]

Immediately after the accident, a faculty specialist team was organized in a wide variety of areas including soil, vegetation, animal life, fishery, forestry, etc. to carry out the research studies, where my role was making up the team and the research projects. It was important that the results of these studies were useful for the recovery of the affected area; so my other role was to work for official announcement of the results. Twelve meetings were held to report research results since November 2011 and an easy-to-understand book was published in Japanese (Nakanishi, 2013). This book was published to allow a wide range of ordinary people to have a correct understanding of the impact of radioactive materials on agriculture. In addition, a collection of papers was published as a series of book (titled Agricultural implications of Fukushima nuclear accident) by Springer in 2013 and in 2016 and they were made available for free download on the web so that the results of the research and studies could be widely shared with foreign and domestic researchers. A follow-up 3rd book is going to be published early in 2017 by Springer.

#### Radioactive cesium distribution in a rice plant

The gaseous fission products released from Fukushima Daiichi nuclear accident was adsorbed on anything when they first touched, which was exposed to the air at the time of the accident. In the case of soil, the soil surface was the most contaminated part and the depth movement of the radioactivity is now about 1–2 mm/year which is the similar to that of the Chernobyl accident. About a month later, radioactive cesium (^137^Cs + ^134^Cs) was found to be the main radioactive nuclides remained in the environment. Therefore, the study was focused on the movement of radiocesium.

**Fig. 30:**
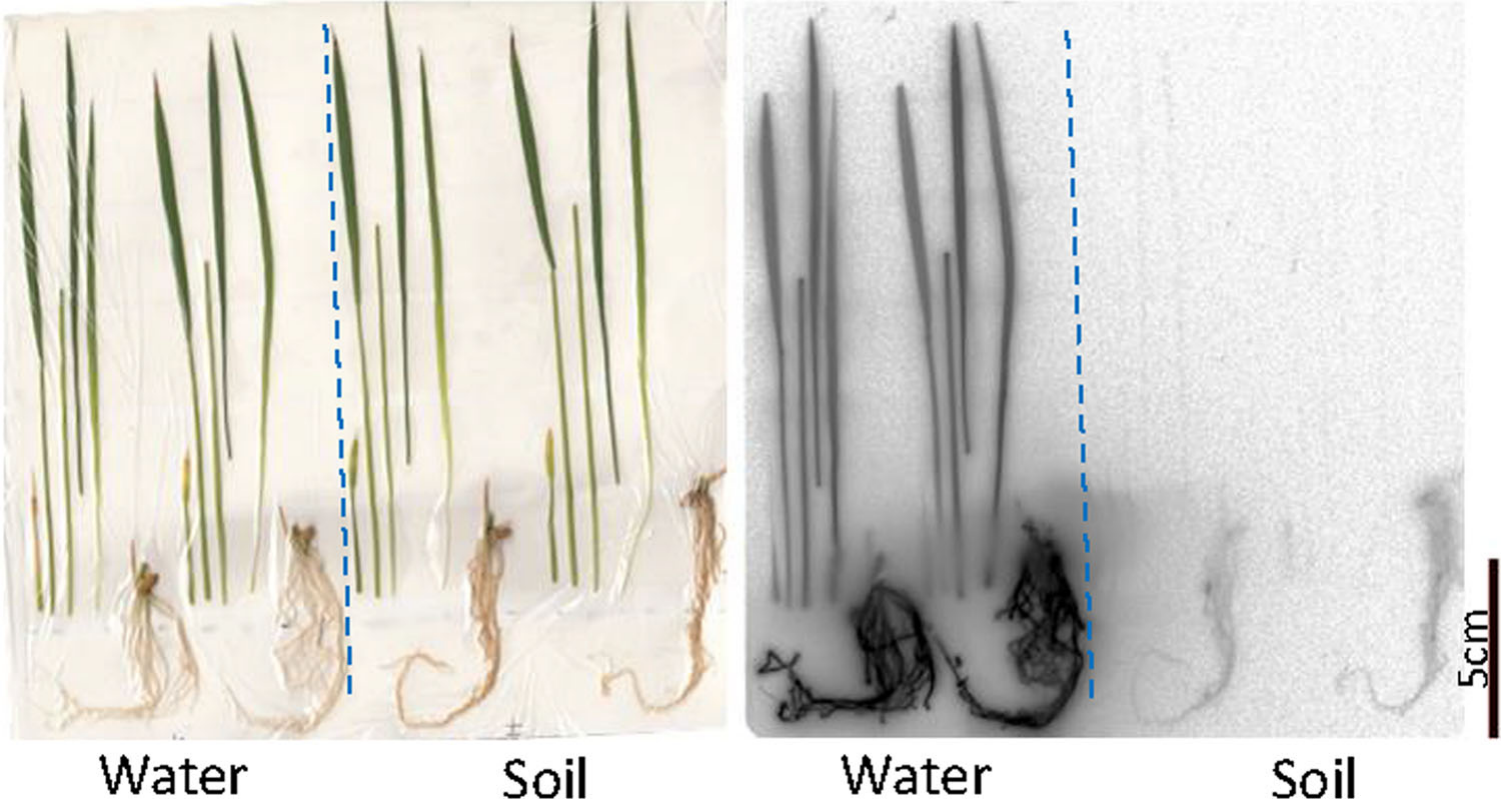
Real-time imaging of ^137^Cs in a rice plant. *Note* Rice plants were grown separately in water and soil cultures. *Left* picture; *right* RI image. After 24 h, the ^137^Cs dissolved in water was taken up by the plant whereas in presence of soil ^137^Cs was firmly adsorbed on to the soil and plants could not absorb much amount of ^137^Cs. Especially, at up-ground part of the plant grown in soil, hardly any ^137^Cs distribution was observed

Since a lot of rice is produced in the Fukushima Prefecture, one of the main concerns of the people was how much radiocesium is absorbed by the rice plant. Applying K to the farming land was an excellent measure to reduce the absorption of radiocesium by the plant. Since Cs and K can behave in a similar fashion, any possible difference was studied in detail using ^137^Cs and ^42^K produced from the ^42^Ar generator. Real-time RI imaging system showed that when the rice is grown in contaminated soil, most of the radiocesium adsorbed on the soil was not picked up by the rice plants; this was different from the result of water culture where most of the radiocesium in culture medium was absorbed by the plant. Figure 30 shows the difference between the radiocesium uptake manner between water and soil cultures.

#### Micro-autography (MAR)

**Fig. 31:**
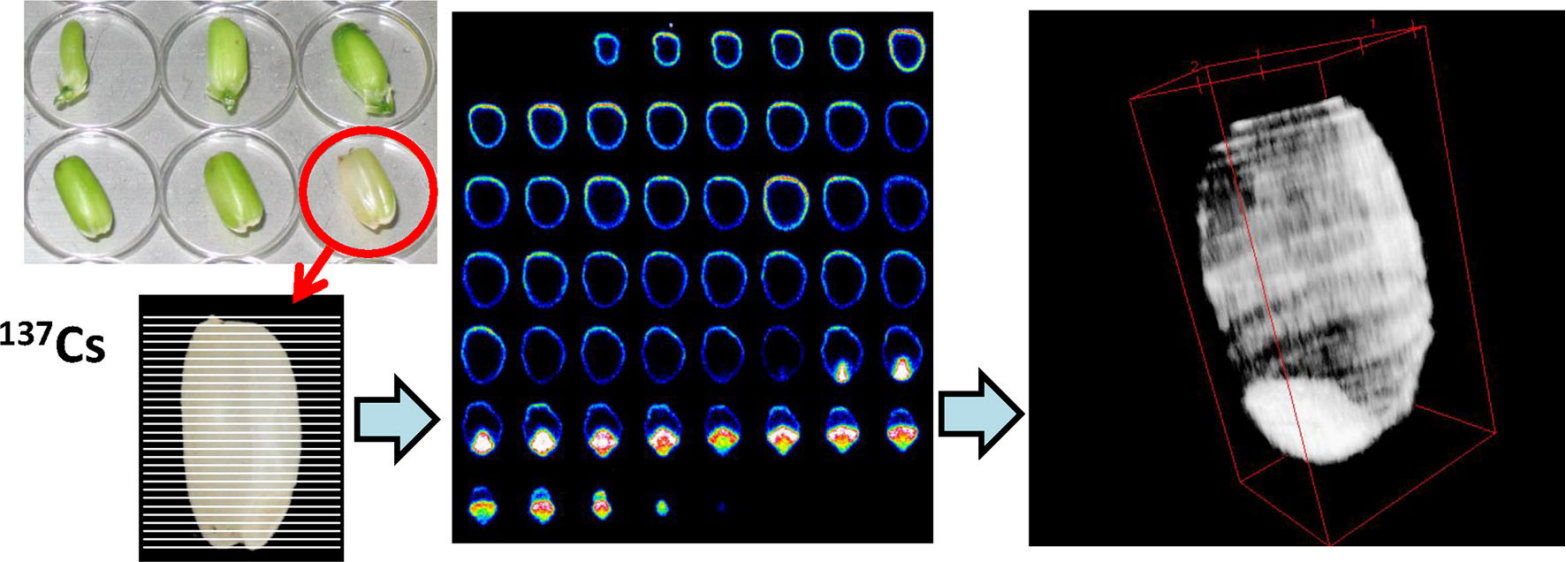
^137^Cs distribution in a rice grain. After 15 days of flowering, the rice grain was harvested from the plant to which ^137^Cs was added during the growth. The brown rice was sliced and placed on an imaging plate. Then, all the images of the sliced sections were used to construct 3-dimentional distribution of ^137^Cs. The distribution of ^137^Cs was high at the embryo and the surface of the brown rice

One of the important objectives of our study was to find out how and at what site the radioactive cesium was accumulated in the rice grain using radioactive tracer ^137^Cs. After supplying the ^137^Cs, the rice grain during the developmental stage was harvested and the distribution of ^137^Cs was recorded. Figure 31 shows the trial to construct a three dimensional image of ^137^Cs in a brown rice gain 15 days after flowering. Sliced grain was placed on an imaging plate for exposure and a three dimensional image was constructed from all these images. It is evident from the figure that ^137^Cs was distributed in the embryo and the periphery of endosperm; outer skin of the grain was removed as bran when milled.

**Fig. 32:**
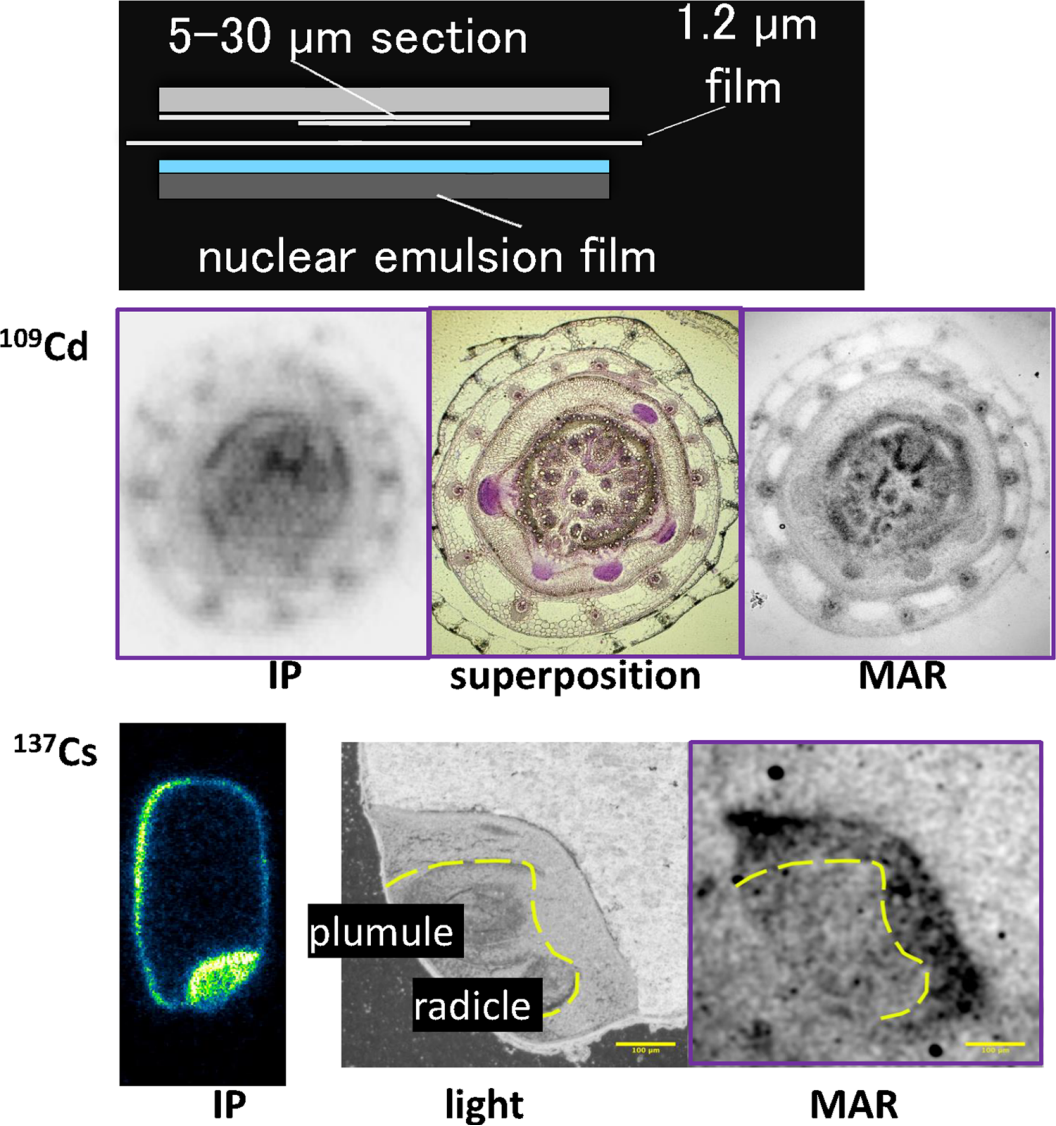
Development of micro-autoradiography. *Note* To get higher resolution of the image than that of an imaging plate, micro-autography method was developed. Nuclear emulsion film was prepared and placed at the sample. *Middle figure* is an example of ^109^Cd distribution image in the connection part between roots and stem. The *left image* was ^109^Cd distribution obtained by an imaging plate; the *right image* was by micro-autoradiography, and when it was superimposed to the *light image* the figure obtained is shown in the middle. The *bottom images* are ^137^Cs distribution in a rice grain. In micro-autoradiography, ^137^Cs was found to be distributed around the plumule and radicle in embryo

To examine the ^137^Cs accumulated part in more detail, a micro-autoradiography (MAR) method was developed, where nuclear emulsion film was prepared and attached to the thin section of the sample (Fig. 32). The figures in the middle show the comparison of ^109^Cd distribution image obtained by an imaging plate (IP) to that by MAR at the connection part of the root and up-ground part in a rice plant demonstrating that MAR provides an image with higher resolution. In the case of ^137^Cs distribution of the rice grain, the accumulation site was also studied. Although ^137^Cs was accumulated at embryo indicated by an IP, ^137^Cs concentration was low at the specific site of plumule and radicle, and the surrounding part of these tissue contained higher amount of ^137^Cs.

## Conclusions

Application of radiation and radioisotopes provide new findings what no other methods could do. Especially, in the case of water, only neutron beam provides the water specific image and for water movement itself; without using ^15^O it is hardly possible to calculate the small amount of water.

The relationship between the element and the plant is one of the important issues, since plants live on inorganic elements. However, the study of the element whose radioactive isotope is not available has not been developed well. For example, the research on B or Si has been far behind compared to the other elements such as P or S. Therefore, it is very important to prepare and apply the radioisotopes for research which are not commercially available even if they have relatively short half-lives, such as ^15^O, ^38^ Mg and ^42^K.

There are many advantages of using radioactive tracers especially for the real-time imaging. Image is the direct evidence to show the activities of living plants. When RI imaging is referred, only static imaging using an imaging plate is widely performed and the real-time imaging using the conventional radioisotopes has not been studied well. The wide application of RI imaging is expected not only in plant study but also for foods, industrial materials, etc.

In the case of Fukushima, after the accident the local government had set up the system to measure the radioactivity of all agricultural products. In the case of rice, radioactivity of all the rice grains produced in Fukushima was measured prior to marketing. Now no bag was found showing higher radioactivity than the regulation value of 100 Bq/kg. However, since the half-life of ^137^Cs is 30 years, we still have to continue the study to understand the radio-contamination effect in agricultural environment including forests.
